# Modeling Zeta Potential for Nanoparticles in Solution: Water Flexibility Matters

**DOI:** 10.1021/acs.jpcc.2c08988

**Published:** 2023-05-09

**Authors:** Paulo Siani, Giulia Frigerio, Edoardo Donadoni, Cristiana Di Valentin

**Affiliations:** † Dipartimento di Scienza dei Materiali, Università di Milano Bicocca, via R. Cozzi 55, 20125 Milano, Italy; ‡ BioNanoMedicine Center NANOMIB, University of Milano-Bicocca, 20126 Milano, Italy

## Abstract

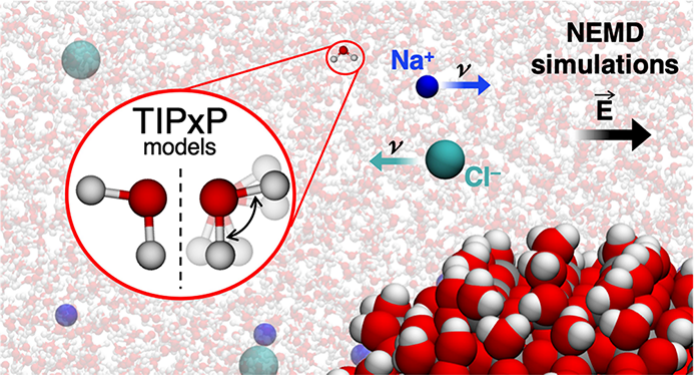

Nonequilibrium molecular dynamics simulations were performed to study the electrokinetic properties of five mainstream TIP*x*P water models (namely, TIP3P-FB, TIP3Pm, TIP4P-FB, TIP4P-Ew, and TIP4P/2005) in NaCl aqueous solutions in the presence of a negatively charged TiO_2_ surface. The impact of solvent flexibility and system geometry on the electro-osmotic (EO) mobility and flow direction was systematically assessed and compared. We found that lack of water flexibility decelerates the forward EO flow of aqueous solutions at moderate (0.15 M) or high (0.30 M) NaCl concentrations, in some special cases to such an extent that EO flow reversal occurs. Zeta potential (ZP) values were then determined from the bulk EO mobilities using the Helmholtz–Smoluchowski formula. The straight comparison against available experimental data strongly suggests that water flexibility improves the ZP determination of NaCl solutions adjacent to a realistic TiO_2_ surface under neutral pH conditions.

## 1 Introduction

With the vast and rapidly growing use of nanotechnology in strategic sectors of society, the molecular understanding of its fundamental ingredient, nanoparticles (NPs), is of utmost importance to achieve satisfactory outcomes in real-world applications. Of most practical relevance for micro and nanosized particle research is the electrokinetic properties of NPs in solution, which demand state-of-the-art methods to quantify them accurately. In the last decade, nonequilibrium molecular dynamics (NEMD) methods have arisen as a powerful tool to do this job, bringing about a better understanding of fluids interfacing with inorganic surfaces under the action of an external electric field.^1−4^ However, the widespread use of NEMD methods to study the electrokinetic phenomena of NPs in solution also brings with it the need for best practice protocols to achieve reliable and accurate simulation outcomes. Hence, systematically benchmarking the transferability of standardized simulation setups and mainstream force fields (FFs) to unusual system compositions (e.g., bioinorganic nanoparticles in aqueous solution) beforehand, although a time-consuming task, must be done to ensure the simulation results’ reliability.

With that in mind, one can determine the reliability and accuracy of FF potentials as well as the influence of different simulation setups by assessing their performance in describing the system’s properties of interest, which commonly resorts to often-reported experimental measurements as target data. Among the most reported experimental quantities of NPs in solution are the NPs’ electrokinetic mobility and its associated electrostatic potential at the surface of shear, widely known as zeta potential (ζ potential, ZP). ZP is a key physicochemical property of the system that controls the NP behavior in solution (e.g., dispersion and stability). Electrophoretic light scattering (ELS),^5,6^ and, more recently, electroacoustics^7^ have become the experimental methods of choice to quantify ZP in diluted and concentrated dispersions, respectively. However, the translation of NPs’ mobility to ZP is not straightforward and relies on the appropriate theoretical treatment according to the NPs’ physical nature (e.g., size and curvature). Thus, theoretical and experimental studies of NPs in solution have been more and more synergically bridged by atomistic NEMD simulations, allowing an in-depth understanding of the molecular origins of ZP.^3,8−10^

In that vein, a seminal study by Předota and co-workers^1^ has made a step toward a more precise NEMD simulation framework to determine ZP from limiting electro-osmotic (EO) mobility, overcoming some previous methodological issues in exactly defining the surface of shear in solid/liquid interfacial systems. This approach showed excellent performance in studying the impact of pH and salt concentration on the ZP of TiO_2_ and SiO_2_ surfaces interfacing an aqueous medium. Nevertheless, the NEMD predictions of positive ZP values for TiO_2_ surfaces at neutral pH and high salt concentration, although predicting the correct trend for the ZP values, differed qualitatively from those found experimentally (negative ZP values) under similar solution conditions.^11^ The authors interpret this discrepancy due to the occurrence of charge inversion and EO flow reversal mainly driven by strong adsorption of cations on the negative TiO_2_ surface, invariably yielding counter-intuitive positive ZP values.

However, not only the intrinsic physicochemical properties of a particular system composition but also the solvent modeling influences the electrodynamic properties of bulk-like aqueous solutions. Previous NEMD simulation reports found that collective transport coefficients, such as shear and bulk-water viscosity, are in greater agreement with experimental measurements when intramolecular flexibility is incorporated into the solvent modeling.^12^ Moreover, Wallqvist and Teleman^13^ demonstrated that the intramolecular flexibility of water molecules significantly impacts their bulk properties. They found that incorporating intramolecular motion in water molecules impacts their dynamic properties, slowing down the overall fluid diffusion due to enhanced dipole–dipole coupling between water molecules and their surrounding fluid. Furthermore, previous NEMD simulation studies suggest that water flexibility does not substantially impact the thermal conductivity of water,^14^ and either flexible or rigid water models can capture the overall experimental behavior of thermal conductivities under high-density and -temperature conditions.^15^

Rigid water models are widely adopted in the determination of ZP using NEMD simulations^1−3,10,16^ and very often NEMD output information (e.g., ion distribution, EO velocities) is utilized as reference and/or input data to validate and/or complement analytical models.^2,16−19^ However, to the best of our knowledge, no studies to date have systematically investigated the impact of water flexibility on the EO flow and electrokinetic potential (ZP) of aqueous solutions at varying salt concentrations interfacing with realistic inorganic surfaces. To fill this gap, we benchmark five TIP*x*P water models (labeled here as TIP3P-FB, TIP3Pm, TIP4P-FB, TIP4P-Ew, and TIP4P/2005) together with their ad hoc parametrized monovalent ions (Na^+^, Cl^–^) to predict the electrokinetic mobilities and the ZP of aqueous solutions at 0.15 and 0.30 M of NaCl sandwiching an anatase (101) TiO_2_ slab under neutral pH conditions. TIP3P-FB corresponds to the three-site TIP3P water model optimized by the ForceBalance method,^20^ TIP3Pm stands for the three-site TIP3P model modified for usage in combination with the CHARMM FF, and commonly adopted in biomolecular simulations,^21,22^ TIP4P-FB refers to the four-site TIP4P model parametrized by the ForceBalance method,^20^ TIP4P-Ew is the four-site TIP4P model re-parametrized for use with standard Ewald summation,^23^ and TIP4P/2005^24^ is the four-site TIP4P model combined with the Madrid 2019 model, which uses scaled charged for ions.^25^

The paper is organized as follows: first, we examine the ion distribution normal to the negatively charged TiO_2_ surface (Section 3.1). In Section 3.2, we evaluate the impact of different simulation setups (e.g., incorporating water flexibility and slab correction) on the EO mobility of water molecules and surface charge screening normal to the TiO_2_ slab. Finally, we determine the ZP values of the negatively charged TiO_2_ surface from the limiting EO mobilities by applying the Smoluchowski theory in Section 3.3. Then, we assess the accuracy of our NEMD simulations in predicting ZP values in light of available experimental data for anatase TiO_2_ NPs under close solution conditions of pH and salt concentration.^11^

## 2 Computational Details

**Scheme 1:**
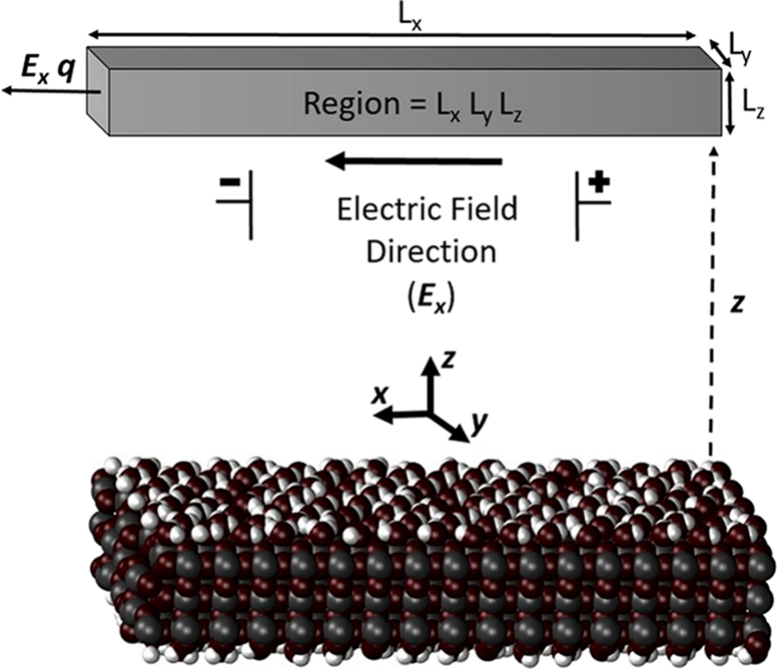
Schematic Representation of the TiO_2_ Slab, the Applied Electric Field, and the Region Used to Average Water EO Mobilities

Herein, we used NEMD simulations to estimate the ZP of a hydroxylated TiO_2_ flat surface under explicit water solvation, which is a fair approximation to microsized TiO_2_ particles in solution. The hydroxylated TiO_2_ flat surface was modeled with a TiO_2_ (101) anatase slab of 768 TiO_2_ units (three triatomic layers) placed into an orthorhombic supercell of 60.8273 Å × 41.9119 Å × 115.0000 Å. Molecularly adsorbed water and hydroxyl groups were attached to all 5-fold coordinated Ti atoms on the TiO_2_ (101) anatase surface in a ratio of 70/30, respectively. The FF for the hydroxylated anatase TiO_2_ atoms is taken from Rouse et al.,^26^ who recently parametrized this set of empirical FF parameters in a bottom-up fashion to reproduce ab initio reference data and classically describe anatase TiO_2_ nanoparticles interfacing water and small biomolecules. It is noteworthy that each hydroxyl group bonded to the 5-fold coordinated sites (which become 6-fold coordinated Ti sites) bears a net charge of −0.4 *e* and such setup yields a surface charge density equal to −0.62 *e*/nm^2^ mimicking that found in experimental measurements for TiO_2_ nanoparticles under neutral pH conditions.^27^ The resulting negative charge of −16 *e* on the TiO_2_ surface is counterbalanced with the appropriate number of Na^+^ counter-ions, and the appropriate number of cations and anions is added to set the salt concentration at 0.15 or 0.30 M of NaCl in solution. The EO flow is driven by an external electric field of 0.02 V/Å applied in the *x*-direction (Scheme 1) tangentially to the TiO_2_ slab. To check whether the choice of the electric field strength has considerably impacted the ion distribution, we estimate the number density profile of Na^+^ ions normal to the TiO_2_ surface in the absence of an electric field at 0.30 M of salt concentration (for details please see Section S1, Supporting Information). The equilibrium MD (EMD) simulation is then compared against the ion distribution of Na^+^ estimated for the same system and simulation setup, although under an electric field strength of 0.02 V/Å. We found that, for the physicochemical nature of the system under study here (negatively charged TiO_2_ surface interfacing with an aqueous solution rich in NaCl salt), where a strong adsorption tendency of Na^+^ particles over the TiO_2_ surface is observed, the chosen electric field strength has little impact on the ion distribution compared to the EMD predictions under the same conditions. Nonetheless, one should be aware that the decision on the electric field strength is case-dependent and hence, shall be verified beforehand.

The NEMD simulations were extended up to 120 ns in the NVT ensemble at *T* = 303.15 K using the LAMMPS simulation package (version 29 Oct 2020), and the last 20 ns of MD production were used for data analysis. The temperature was held constant using a Langevin friction force with a damping coefficient of 0.1 ps^–1^. The long-range solver PPPM^28^ handled the electrostatic interactions with a real-space cutoff of 10 Å and a threshold of 10^–5^ for the error tolerance in forces. For the short-range Lennard–Jones 12–6 potential, we utilized a cutoff of 10 Å with a switching function applied beyond 8 Å. Newton’s equations of motion were integrated in time using the Velocity–Verlet integrator with a timestep of 1 fs. The SHAKE^29^ algorithm was used to impose holonomic constraints on all covalent bonds involving hydrogen atoms.

If not stated otherwise, the EO mobility of water molecules parallel to the applied electric field (Scheme 1) was collected on-the-fly along the *z*-direction normal to both (equivalent) sides of the TiO_2_ slab in 0.1 Å-wide bins along the *z*-direction and then averaged out over the entire 20 ns-long MD production phase. To prevent any possible artifact coming from the finite-size z-boundary and then avoid any influence on the zeta potential estimation due to the deviation of ion and water densities from their bulk behavior near the z-boundaries, we exclude any contribution to the averaged water mobility coming from the region where both ion and water density deviates from its bulk-like behavior (between 50 and 60 Å from the TiO_2_ slab, Section S2 of Supporting Information). Thus, only the EO mobilities of water molecules far enough from the TiO_2_ surface within Region = *L_x_L_y_L_z_* in Scheme 1 (between 40 and 50 Å from the TiO_2_ slab center), thus representing the bulk-like EO mobilities, were taken and averaged out. Five mainstream TIP*x*P water models were studied, namely, TIP3P-FB,^20^ TIP3Pm,^21,22^ TIP4P-FB,^20^ TIP4P-Ew,^23^ and TIP4P/2005^24^ with their respective ad hoc parametrized Na^+^ and Cl^–^ ions: TIP3P-FB,^30^ TIP3Pm,^31,32^ TIP4P-FB,^30^ TIP4P-Ew,^33^ and Madrid 2019 model for TIP4P/2005.^25^ The complete list of empirical FF parameters for the water models and their corresponding monovalent ions studied herein are reported in Table 1. It is important to note that these acronyms refer to the above-specified combination between water and ion FFs throughout the text.

**Table 1:** Set of Empirical FF Parameters for the Five Water Models and their Ad Hoc Parametrized Monovalent Ions (Na^+^, Cl^–^) with the Cross-Interaction LJ Parameters for Unlike Atoms Obtained from the Lorentz–Berthelot Mixing Rules

model	TIP3P-FB	TIP3Pm	TIP4P-FB	TIP4P-Ew	TIP4P/2005
*q*(O) (*e*)	–0.84844	–0.834	–1.05174	–1.04844	–1.1128
*q*(H) (*e*)	+0.42422	+0.417	+0.52587	+0.52422	+0.5564
ε(OO) (kcal/mol)	0.15587	0.1521	0.17908	0.16275	0.1852
σ(OO) (Å)	3.1780	3.1507	3.1655	3.16435	3.1589
ε(HH) (kcal/mol)	0.0	0.0460	0.0	0.0	0.0
σ(HH) (Å)	0.0	0.4000	0.0	0.0	0.0
*r*(O–M)^a^ (Å)			0.10527	0.125	0.1546
*r*_0_(O–H) (Å)	1.0118	0.9572	0.9572	0.9572	0.9572
θ(H–O–H) (Å)	108.15	104.52	104.52	104.52	104.52
*k*(H–O–H) (kcal/mol/Å^2^)	55^b^	55	55^b^	55^b^	55^b^
ε(Na^+^Na^+^) (kcal/mol)	0.02759452	0.0469	0.02499594	0.02154025	0.35190153
σ(Na^+^Na^+^) (Å)	2.600	2.51367	2.580	2.552	2.21737
ε(Cl^–^Cl^–^) (kcal/mol)	0.63803333	0.1500	0.64716164	0.65269755	0.01838504
σ(Cl^–^Cl^–^) (Å)	4.098	4.04468	4.121	4.136	4.69906

a r(O–M) indicates the displacement between the O position and the massless particle M position.

b Assigned by analogy with the flexible version of the TIP3Pm water model.

All water models mentioned above were originally parametrized to be used as rigid molecules, so the bond length and angle were held fixed at their respective equilibrium values (Table 1); they are tagged as “rigid models” throughout the text. Furthermore, we introduced bending degrees of freedom in the rigid models to model their flexible version. Thus, the H–O–H angular bending around its standard equilibrium bond angle value was allowed; these water models are referred to as “flexible models” throughout the text. For the sake of comparison, the transferability of angle bending is assumed among the water models, and the angular force constant is taken from the flexible version of the TIP3m water model. The parametrization and optimization of angular force constants for the rigid water models are not in the scope of this work. For the TIP3Pm water model, the NBFIX correction is applied to the cross-interaction between Na^+^ and Cl^–^, with ε(Na^+^Cl^–^) = 0.083875 kcal/mol and σ(Na^+^Cl^–^) = 3.324 Å.

All system models are composed of Na^+^ and Cl^–^ ions symmetrically distributed in an aqueous slab 57.5 Å thick placed above and below the hydroxylated TiO_2_ surface. Widespread methodologies to solve long-range electrostatics in typical 3D periodic systems, such as Particle Mesh Ewald (PME) and Particle-Particle Particle-Mesh (P3M), are not readily applicable to 2D periodic systems. One long-standing methodological issue for 2D periodic systems carrying charged particles lies in the accurate and efficient treatment of long-range forces. Thus, several approaches have been proposed to approximate long-range interactions for these systems, such as the 2D Ewald summation method by Parry,^34^ the use of empty layers between slabs combined with 3D Ewald summation by Spohr,^35^ and lately, the removal of spurious multipole inter-slab interactions using a correction term for the 3D Ewald method by Yeh and Berkowitz.^36^ Due to enhanced performance and fair accuracy over the 2D Ewald method, the latter was our method of choice, although implemented for the 3D P3M solver instead of the 3D Ewald one. In practice, the slab correction implemented in the P3M solver dumps out any spurious inter-slab interactions and requires the insertion of an empty volume between the slabs at least three times larger than the unit cell dimension in *z*.

**Scheme 2:**
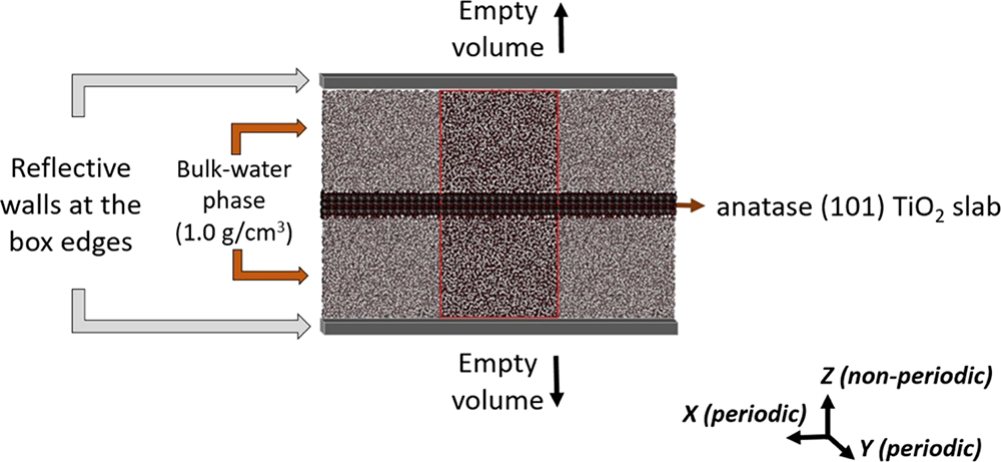
Maintenance of the Symmetrical Arrangement of Ions via Reflective Walls Using the P3M Solver with Slab Correction for the Treatment of Long-Range Electrostatic Forces Normal to the Anatase (101) TiO_2_ Surface (*z*-Direction) The edges of the central simulation box (front view, XZ plane) are delimited by red lines.

**Scheme 3:**
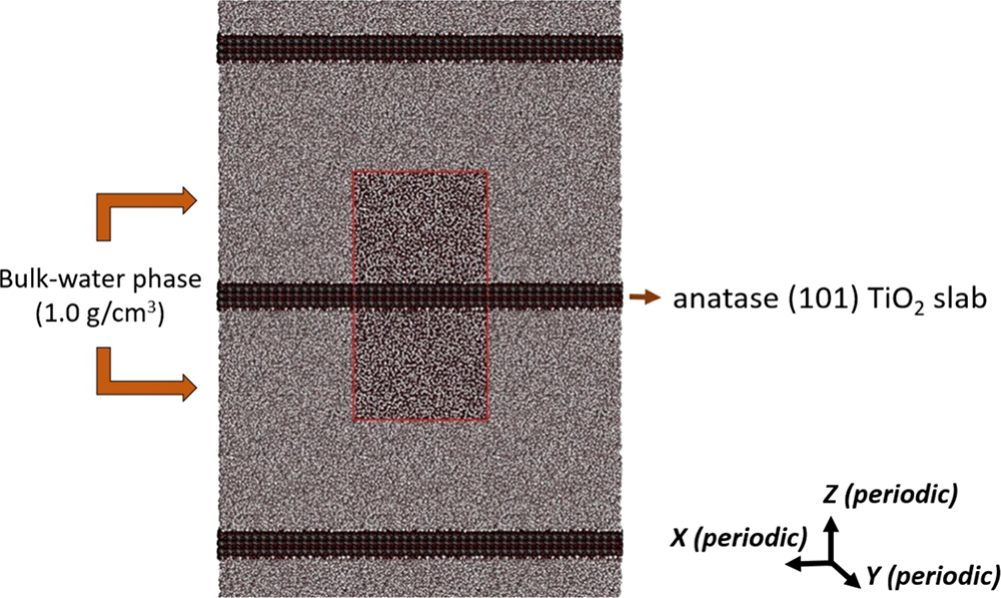
Full-3D Periodicity (Full-3D PBC) and Use of Standard P3M Solver for the Treatment of Long-Range Electrostatic Forces Normal to the Anatase TiO_2_ Surface (*z*-Direction)^a^ The edges of the central simulation box (front view, XZ plane) are delimited by red lines and replicated in all three dimensions. Water molecules and ions can unrestrictedly migrate through the PBC edges, and no SC correction is applied to solve the electrostatic forces normal to the TiO_2_ slab.

Two different MD simulation setups are investigated:

(I) The TiO_2_/water interface is described with an approximated 2D slab model (water/TiO_2_ slab/water), and the primary cell (60.8273 Å × 41.9119 Å × 115.0000 Å) is extended in the *z*-direction perpendicular to the TiO_2_ slab with an empty volume resulting in a *z*-dimension three times larger than the primary one. The arrangement of ions above and below the TiO_2_ slab is symmetrical and is maintained over the NEMD simulations through a reflective wall positioned at the edges of the simulation box in the *z*-direction. The use of reflective walls keeps the particles in the original box before the P3M expansion, therefore preventing particle migration through the *z*-boundaries and extra computational workload. When a particle moves through these walls on a specific timestep by a certain distance Δ*x*, the same particle is put back inside the unit cell at a Δ*x* distance from the wall with its velocity and force components having the sign flipped. We found that reflective walls have negligible impact on the water and ion distribution at the *z*-boundaries in the P3M-SC setup proposed in this work. Further details of the influence of reflective walls on the water and ion distributions at the *z*-boundaries can be found in Section S2, Supporting Information. The Particle-Particle Particle-Mesh electrostatic solver is utilized (P3M) and slab correction (SC)^36^ is turned on, so that inter-slab interactions of the residual dipole of the system in the *z*-direction are removed and slab-slab interactions are effectively turned off. We refer to this MD setup with the label “P3M-SC” through the text and its schematic representation is given in Scheme 2.

(II) No reflective walls are added at the edge of the simulation box and the regular Particle-Particle Particle-Mesh (P3M) electrostatic solver (without slab correction) is applied in all three dimensions, so water molecules and ions can migrate through the boundaries of the simulation box. This MD setup is labeled as “P3M” through the text and its schematic representation is given in Scheme 3.

**Scheme 4:**
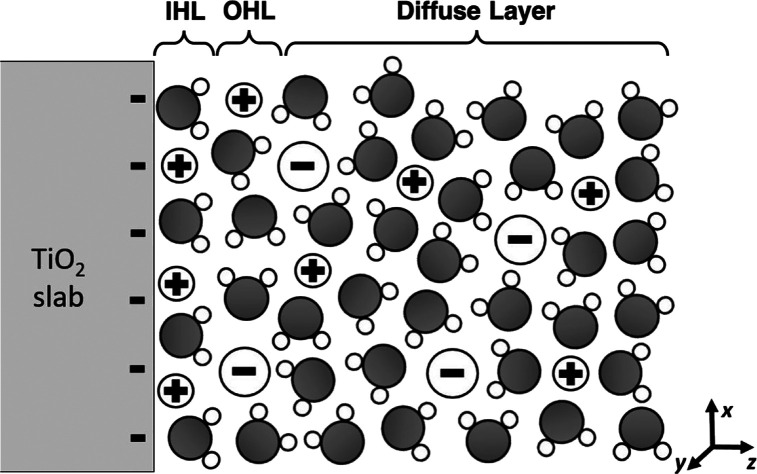
Schematic of the Electrical Double Layer Surrounding a Negatively Charged Particle in Bulk Water Phase as Described by Grahame’s Model: In Order of Distance from the TiO_2_ Surface, IHL, OHL, and Diffuse Layer

A simplified representation of the electrical double layer at the negatively charged TiO_2_ surface can be found in Scheme 4. Cations form a first-ordered layer on the TiO_2_ surface to neutralize and screen its negative charge, which is known as the inner Helmholtz layer (IHL). The next ordered layer is called the outer Helmholtz layer (OHL) and the outer solution layer, where ions and water molecules diffuse freely, is called diffuse layer.

The influence of water flexibility on some fundamental solution properties, namely, static dielectric, viscosity, and cation-anion structure was investigated by means of several equilibrium MD simulations (EMD) of each water model considered in this work, using either their flexible or rigid version: one set of EMD simulations considered a simulation box made up of pure water to estimate the static dielectric of the aqueous medium and a second set of EMD simulations of aqueous salt solution at 0.15 M of NaCl to unveil the viscosity and cation-anion structure predicted by the different water/ion FFs adopted in this work. Thus, we have built simulation boxes of 50 × 50 × 50 Å^3^ filled with TIP3Pm, TIP3P-FB, TIP4P-FB, TIP4P-Ew, or TIP4P/2005 at the initial density of 0.99 g/cm^3^. For the aqueous salt solutions, we added the proper number of Na^+^ and Cl^–^ ions to set the salt concentration at 0.15 M. The production phase was extended up to 100 ns using an NPT ensemble at 1.0 atm in all EMD simulations, from which the last 20 ns are used to predict the cation-anion RDF profiles (Figure S3). For theoretical background on viscosity and dielectric constant calculations, please see Section S10, Supporting Information.

## 3 Results and Discussion

### 3.1 Ion Distribution

First, we examine how different water/ion FFs (TIP3P-FB, TIP3Pm, TIP4P-FB, and TIP4P-Ew), using or not flexible water molecules as well as full-3D periodic or 2D slab models (P3M or P3M-SC, respectively) affect the ion distribution normal to the negatively charged TiO_2_ surface.

**Figure 1:**
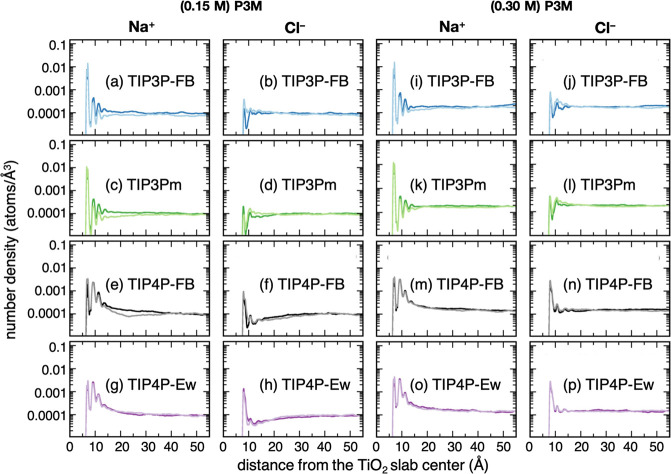
Number density profiles of co- and counter-ions normal to the negatively charged TiO_2_ surface at 0.15 M (a–h) or 0.30 M (i–p) NaCl concentration under regular P3M treatment. Color codes: (blue) TIP3P-FB and flexible model, (light blue) TIP3P-FB and rigid model, (green) TIP3Pm and flexible model, (light green) TIP3Pm and rigid model, (black) TIP4P-FB and flexible model, (gray) TIP4P-FB and rigid model, (violet) TIP4P-Ew and flexible model, and (light violet) TIP4P-Ew and rigid model.

Figure 1 shows the number density profiles for TIP3P-FB, TIP3Pm, TIP4P-FB, and TIP4P-Ew FFs averaged in 0.1 Å-wide bins normal to the TiO_2_ slab under regular P3M treatment at 0.15 and 0.30 M.

Analysis of Figure 1 reveals that TIP3P FFs induce a more extensive Na^+^ adsorption on the TiO_2_ surface than what we observe in the case of TIP4P-Ew and TIP4P-FB (Figure 1a,c vs e,g; Figure 1i,k vs m,o). Indeed, whether TIP3Pm or TIP3P-FB FF are chosen, we notice strong Na^+^ adsorption and the formation of a thin and dense layer on the TiO_2_ surface, while for TIP4P-Ew and TIP4P-FB FFs we see that Na^+^ adsorption happens to a lesser extent on the TiO_2_ surface. Figure 1e,g also shows a larger concentration of Na^+^ ions in the diffuse layer when using TIP4P-Ew and TIP4P-FB FFs: we identify three evenly distributed and well-defined peaks within 7 Å < *z* < 11 Å, in which the first and second peaks are similarly populated. Further probability analysis (Figures S4 and S5, Supporting Information) corroborates the findings above, indicating a higher exchange capacity of TIP4P Na^+^ ions between the structured layer and the bulk-water phase than those modeled by TIP3P FFs. The probability of finding Na^+^ ions near the TiO_2_ at 0.15 M of NaCl in solution is, in ascending order: TIP4P-Ew < TIP4P-FB < TIP3Pm < TIP3P-FB. We find the same trend at 0.30 M of NaCl in solution as that at lower ionic strength, although higher salt concentration shortens the probability in all NEMD simulations (Figures S4 and S5, Supporting Information). One should keep in mind that the applied electric field may affect the analysis of probability for ions in solution, which has not been verified in this work.

When it comes to the Cl^–^ co-ion distributions, we identify an opposite behavior for Na^+^ ions. We notice that TIP4P-Ew and TIP4P-FB FFs yield a higher condensation of Cl^–^ co-ions next to the adsorbed layer of Na^+^ counter-ions than TIP3P FFs, whether the concentration of NaCl in solution is equal to 0.15 M (Figure 1f,h vs b,d) or to 0.30 M (Figure 1n,p vs j,l). Thus, the Cl^–^ coion packing is enhanced near to the TiO_2_ surface using TIP4P-Ew and TIP4P-FB FFs, while the structured Cl^–^ coion layer gets less dense when TIP3P FFs are adopted. In line with the observations above, we observe that the probability of finding TIP4P-Ew and TIP4P-FB Cl^–^ co-ions near the TiO_2_ surface is higher (Figure S5, Supporting Information) than those modeled by TIP3P FFs. This latter observation also indicates a higher exchange rate of anions between the structured Cl^–^ layers and those bulk solvated when TIP3P FFs are adopted.

Of main interest here is the impact of water flexibility on ion distribution. The introduction of intramolecular motion in water molecules affects the Na^+^ and Cl^–^ density normal to the TiO_2_ in opposite manners, and TIP3P-based NEMD simulations (Figure 1a–d,i–l) are more sensible to solvent modeling changes than the TIP4P-Ew and TIP4P-FB ones (Figure 1e–h,m–p). Moreover, we find that the impact of water flexibility on the ion distributions at 0.30 M resembles the one at 0.15 M (dark-color vs light-color profiles in Figure 1). Due to the inclusion of water flexibility, we see a slightly lower content of Na^+^ counter-ions in the Stern layer, while the Na^+^ density in the diffuse layer increases (dark-color number density profiles in Figure 1a,c,e,g,i,k,m,o). Furthermore, water flexibility causes an overall decrease in the Cl^–^ co-ion condensation in the diffuse layer (Figure 1b,d,f,h,j,l,n,p). This trend is also confirmed by the probability analysis of Na^+^ ions near the TiO_2_ surface (Figure S4): flexible water molecules yield a lower probability of finding Na^+^ counter-ions in the Stern layer, suggesting a higher exchange between cations close to the TiO_2_ surface and those solvated in bulk-water. Also, we find a faster decay in the Cl^–^ probability profiles (Figure S5) for the flexible models, and therefore, a lower probability of finding Cl^–^ co-ions in the diffuse layer when the intramolecular bending of water molecules is allowed. The results and trends found for the P3M-SC setup (Figure S6, Supporting Information) resemble those discussed above for the P3M setup and, therefore, are interchangeable.

We also estimate the Na^+^Cl^–^ RDF profiles using the rigid or flexible version for the three- or four-site water models (Figure S3). Upon analysis of Figure S3, we identify that TIP4P-FB and TIP4P-Ew yield to the most structured Cl^–^ first-coordination shell surrounding the Na^+^ ions. Furthermore, we see that TIP3P-based models lead to a lesser structured first-coordination shell of Cl^–^ ions compared to the former TIP4P models. Rather different is the Na^+^Cl^–^ RDF profile of TIP4P/2005, showing the least populated first-coordinated Cl^–^ shell among the water/ion FFs. Higher level ab initio calculations by Cavallari et al.^37^ predicted a first Na^+^Cl^–^ RDF peak intensity of about 10, suggesting that TIP3P-based water/ion FF combinations may provide a better balance in describing the strength of Na^+^Cl^–^ interaction in aqueous medium.

Altogether, we can infer that ion distribution highly depends on the FF choice for water and ions, even when identical water topologies are considered (e.g., TIP3Pm vs TIP3P-FB or TIP4P-FB vs TIP4P-Ew). In contrast, we see that neither the intramolecular potential of solvent molecules nor the system geometry significantly impacts the overall shape of the ion density profiles. Nevertheless, the introduction of water flexibility does impact the co- and counter-ion density in the structured ionic layers formed on the TiO_2_ surface. The presence of water flexibility increases the content of Na^+^ ions in the diffuse layer while it decreases the number of Cl^–^ ions in the same region, being TIP3P-based NEMD simulation more affected by that.

### 3.2 Surface Charge Screening and Electro-Osmotic Mobility

To shed light on the impact of molecular flexibility of solvent molecules, FF choice, and system geometry on the EO mobility and flow direction of bulk-water molecules, we investigate the interplay between the EO mobility and the surface charge screening by the monovalent ions in the aqueous slab adjacent to the negatively charged TiO_2_ surface. The latter descriptor quantifies how the ion distribution predicted by different FFs (Section 3.1) effectively balances out the negative net charge on the TiO_2_ surface toward the bulk-water phase and can be written as
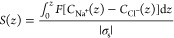
1where *F* is the Faraday constant, σ_s_ is the surface charge, and *C*_Na^+^_ and *C*_Cl^–^_ denote the molar concentration of Na^+^ and Cl^–^, respectively. *S*(*z*) > 1 indicates over-screening of the negative surface charge by excess of positive Na^+^ ions over Cl^–^ ions. *S*(*z*) < 1 indicates sub-screening of the negative surface charge along the *z*-direction. *S*(*z*) equal to the unit indicates that the negative surface charge is completely screened and the concentrations of co- and counter-ions are well-balanced toward the bulk-water phase. Furthermore, the EO mobility of solvent molecules at distance *z* from the surface, μ_*x*_^eo^(*z*), is given by the following relation
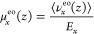
2where ⟨ν_*x*_^eo^(*z*)⟩ is the average EO velocity of the fluid at distance *z* from the TiO_2_ surface, and *E_x_* is the external electric field applied in the *x*-direction (parallel to the TiO_2_ slab).

**Figure 2:**
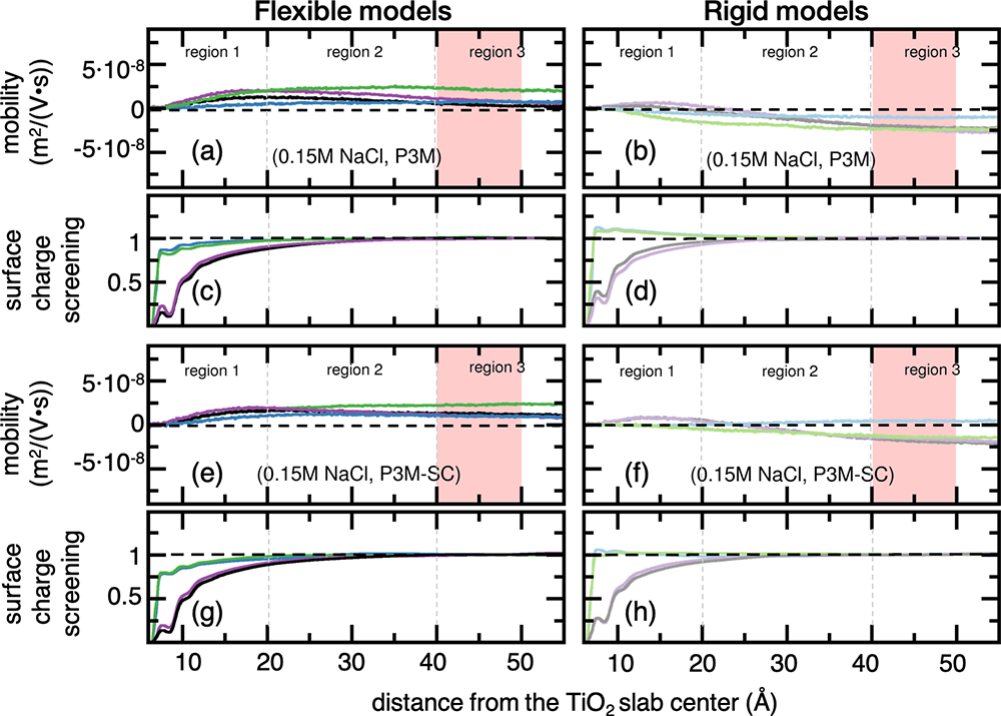
(a, b, e, f) EO mobility and (c, d, g, h) surface charge screening profiles of water molecules normal to the negatively charged TiO_2_ slab at 0.15 M of NaCl in aqueous solution under either regular P3M or P3M-SC electrostatic treatment along the *z*-direction. TIP4P/2005 profiles can be found in Figure S7, Supporting Information. Color code: (blue) TIP3P-FB and flexible model, (light blue) TIP3P-FB and rigid model, (black) TIP4P-FB and flexible model, (gray) TIP4P-FB and rigid model, (green) TIP3Pm and flexible model, (light green) TIP3Pm and rigid model, (violet) TIP4P-Ew and flexible model, and (light violet) TIP4P-Ew and rigid model. The regions in red represent the range where water EO mobilities are averaged for ZP estimation.

**Figure 3:**
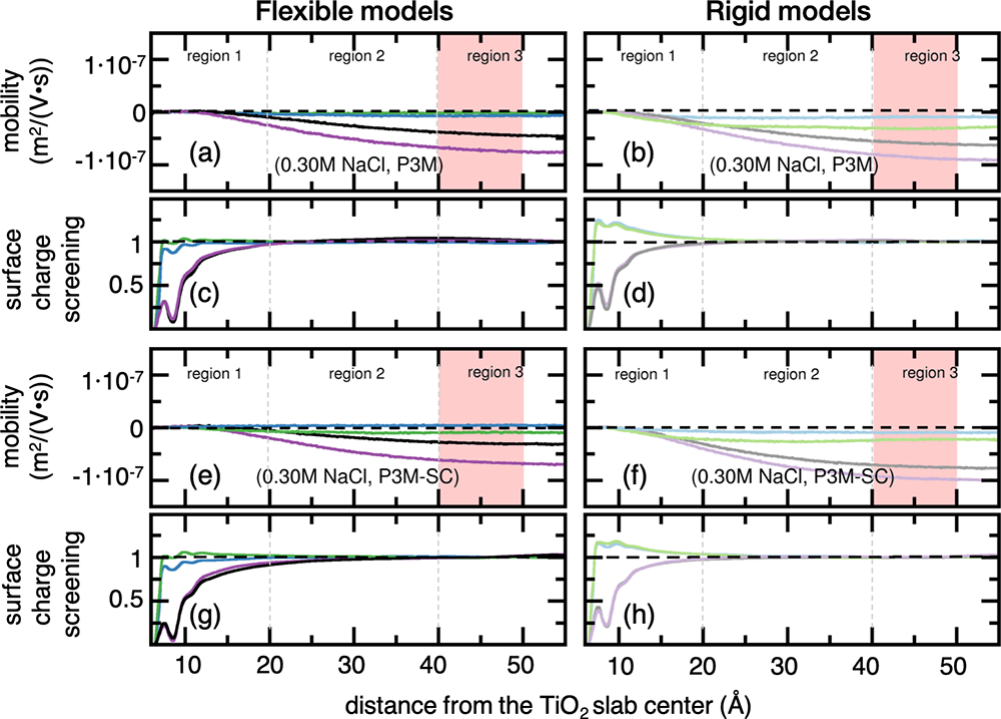
(a, b, e, f) EO mobility and (c, d, g, h) surface charge screening profiles of water molecules normal to the negatively charged TiO_2_ slab at 0.30 M concentration of NaCl in aqueous solution under either regular P3M or P3M-SC electrostatic treatment along the *z*-direction. TIP4P/2005 profiles can be found in Figure S8, Supporting Information. Color code: (blue) TIP3P-FB and flexible model, (light blue) TIP3P-FB and rigid model, (black) TIP4P-FB and flexible model, (gray) TIP4P-FB and rigid model, (green) TIP3Pm and flexible model, (light green) TIP3Pm and rigid model, (violet) TIP4P-Ew and flexible model, and (light violet) TIP4P-Ew and rigid model. The regions in red represent the range where water EO mobilities are averaged for ZP estimation.

Figures 2 and 3 report the *S*(*z*) and the EO mobilities of flexible or rigid water molecules under either the regular P3M or the P3M-SC electrostatic treatment at 0.15 or 0.30 M NaCl, respectively. TIP4P/2005 profiles can be found in Figures S7 and S8, Supporting Information.

For the sake of discussion, we can partition the EO mobility profiles into three distinct regions along the *z*-direction normal to the TiO_2_ surface in Figures 2 and 3: a first region (Region 1) that goes from the TiO_2_ surface up to the end of the well-structured ion layers (at about 20 Å); a transition region (Region 2) starting at 20 Å up to the beginning of the bulk-like solution at about 40 Å; a third region (Region 3) comprehending the bulk-water phase.

Within Region 1, we observe that EO mobilities of flexible water molecules become faster as the distance gets farther from the TiO_2_ surface at 0.15 M of NaCl in solution (Figure 2a,e), and no over-screening takes place (Figure 2c,g). On the contrary, rigid TIP3P water molecules favor EO flow reversal (green and blue lines, Figure 2b,f), and over-screening takes place over the whole Region 1 extending up to the beginning of Region 2 (green and blue lines, Figure 2d,h). Figure 2 also displays a general trend among the simulated systems at 0.15 M of NaCl; lack of intramolecular flexibility always yields a more effective screening of the negative TiO_2_ surface charge (Figure 2d,h). Then, *S*(*z*) profiles rapidly approach the unit and are completely converged within Region 2, while the EO mobility of water molecules undergoes a transition region towards their bulk values. In Region 3, *S*(*z*) reaches the unit and indicates that the surface charge is wholly screened, and co- and counter ions are well-balanced in the aqueous phase. In general, we find that TIP3P- and TIP4P-based FFs provide fair convergence of EO mobility (plateau-like behavior) to their limiting bulk values (Figures 2 and S7).

Upon the addition of salt up to 0.30 M (Figure 3), we notice an enhanced surface charge screening (Figure 3c,d,g,h) compared to the simulated systems at 0.15 M. Also, we observe the discontinuity of EO flow at the solid boundary (solid/liquid interface), which we attribute to the no-slip condition and enhanced shear viscosity at that region (Figure 3a,b,e,f). Furthermore, we find that rigid TIP3P water molecules yield to over-screening in Region 1 (Figure 3d,h). At the very beginning of Region 2, we observe that all EO mobilities are reversed and *S*(*z*) approaches the unit. From that point on, no significant changes occur, and the EO mobilities remain well-converged to their bulk values in Region 3. It is noteworthy that we spot a general trend among all simulated systems; despite the protocol or FF utilized in the NEMD simulations, the rigid models always lead to more effective screening of the total negative charge contained on the TiO_2_ surface (Figure 3d,h vs c,g). Particularly interesting is the correlation between *S*(*z*) and EO mobilities in TIP3P-based NEMD simulations at 0.30 M of NaCl: EO flow reversal is always observed when over-screening occurs. This delicate balance of co- and counter-ions near the TiO_2_ surface dictates the EO flow direction, either forward or reverse, in the case of TIP3Pm or TIP3P-FB, respectively.

Even more impacting on the EO mobility and flow direction is whether intramolecular flexibility is present (or not) in solvent molecules. By eliminating the intramolecular motion of TIP3P- and TIP4P-based water models and making their molecular structure wholly rigid, we identify EO flow reversal in all NEMD simulations (Figure 2a vs b,e vs f), being TIP4P/2005 the only exception (Figure S7). Upon increasing NaCl concentration to 0.30 M, we find that the absence of intramolecular motion of solvent molecules still has a notable influence on EO mobilities, in a similar fashion as that seen at the lower salt concentration (Figure 3a vs b,e vs f). The rigid approximation for water molecules enhances the surface charge screening to such an extent that all NEMD simulations undergo EO flow reversal at such NaCl concentration. There are only two exceptions to this latter observation in which forward EO flow occurs: the combination between flexible TIP3P-FB and P3M-SC treatment (blue curve, Figure 3e) and either rigid or flexible TIP4P-2005 (Figure S8).

When it comes to TIP4P-FB and TIP4P-Ew systems, we observe that lack of water flexibility, among all simulation parameters, has the greatest impact on EO mobility; eliminating intramolecular degrees of freedom of water molecules yields EO flow reversal, whatever the salt concentration, in all these NEMD simulations (violet and black lines in Figure 2a,b,e,f and Figure 3a,b,e,f). Figures 2, 3, S7 and S8 also reveal that TIP4P-based models lack over-screening in Region 1, which can be rationalized on the basis of the interaction strength between Na^+^ and Cl^–^ in solution (Figure S3) and the formation of a densely populated Cl^–^ layer within the OHL (Figure S10).

However, the examination of surface charge screening profiles in Figures 2 and 3, alone, cannot explain the EO flow reversal phenomenon. In a recent study by Rezaei et al., the authors found that EO flow reversal takes place mainly due to the combination of three factors: (i) presence of a stagnant inner Helmholtz layer of adsorbed ions, (ii) charge inversion between the outer Helmholtz and diffusive layers, and (iii) enhancement of the interfacial viscosity.^38^ The authors also point out that, among other parameters, the chosen water/ion FF affects the charge inversion and EO mobility in nontrivial ways.

Thus, we first estimate the fluid viscosity, surface charge screening, and co- and counter-ion distributions for two representative systems, namely, TIP4P/2005 and TIP4P-Ew, which are, respectively, the water/ion FFs showing the most positive and intense EO flow in the electric field direction and the most negative and intense EO flow in the opposite direction of the electric field.

Aware that the surface charge density (−0.62 *e*/nm^2^) in the two above-mentioned systems falls within the regime where a stagnant Na^+^ layer has been observed,^38,39^ we start checking whether over-screening and formation of well-structured ion layers normal to the TiO_2_ slab are present or not. By doing so, we notice that neither the TIP4P-Ew FF nor the TIP4P/2005 FF yield over-screening near the TiO_2_ surface although we identify a significant deviation in the co- and counter-ion distribution among them (Figure S10). To make the interpretation of these data easier, we define the boundaries of the IHL and OHL to coincide with the peaks in the number density profiles of water molecules according to Grahame’s model interpretation in ref (38) (also depicted in Scheme 4). We observe the formation of three well-defined Na^+^ RDF peaks on the TiO_2_ surface in the case of TIP4P-Ew: a first highly populated Na^+^ layer formed in the IHL (at about 7 Å); a second Na^+^ layer formed in the OHL (at about 9 Å); and a third Na^+^ layer (less populated than the former ones) taking place at the beginning of the diffuse layer. For TIP4P/2005, the first Na^+^ RDF peak is absent, while the second and the third resemble those observed for TIP4P-Ew. However, the absence of over-screening can be only explained considering the co-ion distribution and the strength of co- and counter-ion interaction given by the water/ion FFs (see also Section S3). For TIP4P-Ew, we find that the OHL is densely populated by Cl^–^, which effectively counter-balances the accumulated positive charge within OHL, therefore, preventing over-screening of the negative net charge between the OHL and diffuse layer, while, for TIP4P/2005, the accumulation of co-ions in OHL is reduced and a loosely structured Cl^–^ layer is observed, which is likely due to underestimated Na^+^–Cl^–^ interactions found in this water/ion FF.

Furthermore, we investigate how the relative viscosity (η/η_bulk_) is affected when TIP4P/2005 or TIP4P-Ew salt solutions interface a negatively charged TiO_2_ surface by means of the periodic perturbation method (Section S10). To do so, we estimate the fluid viscosity (η) of the whole fluid phase in contact with the TiO_2_ surface and the corresponding bulk viscosity (η_bulk_) calculated in a separate simulation of the same solution without the TiO_2_ slab (Section S7, Supporting Information). In both systems, whether water flexibility was present or not, we found a considerable enhancement of the relative viscosity when the negatively charged TiO_2_ surface is present (η/η_bulk_ ∼ 5–7), with TIP4P-Ew always yielding higher η/η_bulk_ values (top inset in Figure S10) than TIP4P/2005. The bulk viscosity values for the TIP4P/2005 and TIP4P-Ew systems are shown in Figure S9. The observations above suggest that the enhanced fluid viscosity, the presence of strongly adsorbed counter-ions in the IHL, and the high density of co-ions in the OHL are determinant factors to explain the lack of over-screening and the observation of EO flow reversal in the TIP4P-Ew and TIP4P-FB systems.

However, the enhanced surface charge screening near the TiO_2_ surface when water molecules lack flexibility is still not clear. Since the electric dipole moment is significantly affected by the presence or absence of water flexibility, we estimate a correlated water property, namely, dielectric constant, for every TIP*x*P water model. Table 2 shows the static dielectric constant for every TIP*x*P model studied here (please see Section S10 for details).

**Table 2:** Comparison of Static Dielectric Constant (ε_r_) for the Different Water Models in Their Rigid and Flexible Version^a^

ε_r_	TIP3P-FB	TIP3Pm	TIP4P-Ew	TIP4P-FB	TIP4P/2005
RIGID	72.6 (71.6)	86.1 (90.3)	55.7 (59.4)	68.7 (72.0)	53.8 (53.5)
FLEXIBLE	100.3 (99.1)	115.0 (127.3)	65.7 (68.9)	81.1 (85.4)	59.8 (59.0)

a The MD simulations were carried out for pure water in NVT ensemble at 303.15 K (EMD simulations in NPT ensemble at 303.15 K and 1.0 atm are shown in parenthesis).

Interestingly, we find that lack of water flexibility always lowers the dielectric of the aqueous medium whatever the water model adopted. Thus, it is reasonable to assume that the lower dielectric constant of rigid models compared to the flexible ones strengthens the electrostatic interactions between ions and TiO_2_ atoms at the interface, and this is likely the reason behind the main differences found between flexible and rigid models. In line with our speculation above, Jan Bonthuis and Netz^40^ have also attributed the enhanced condensation of ions near the surface due to the low effective dielectric constant of interfacial water.

On the whole, we see that water flexibility, FF choice, and system geometry impact, in nontrivial ways, the EO mobility and EO flow direction of water molecules adjacent to the negatively charged TiO_2_ slab. In summary, we can infer that the presence or absence of intramolecular motion in solvent molecules has the greatest effect on the EO mobility of the fluid among all benchmarked simulation parameters. By only switching from a rigid to a flexible model, opposite predictions of EO flow direction could be observed even for systems of identical composition, where the only difference in the simulation protocol is the treatment of the intramolecular potential of water molecules. Also, we find that restraining water’s intramolecular motion considerably impacts the surface charge screening profiles. Regardless of the FF choice or the system geometry adopted, the presence of water flexibility yields a less effective screening of the negative surface charge of the TiO_2_ slab. In other words, we notice that imposing restraints on the intramolecular motion of water molecules ramps up the Na^+^ counter-ion density near the negative charged TiO_2_ surface (as previously seen in Section 3.1), hence, providing enhanced surface charge screening in that region. This latter observation is likely a consequence of the lowering of dielectric constant due to the rigid water-geometry approximation, which in turn probably strengthens the electrostatic interactions at the interface between ions and the TiO_2_ surface atoms.

### 3.3 Estimation of ZP from EO Mobilities

It is of practical interest to estimate the electrostatic potential difference between the dynamic and the stationary solution layer (slipping plane) near the TiO_2_ surface, the so-called ZP. Several attempts have been made to identify and define this idealized plane, and straight estimations of ZP from electrostatic potential profiles have been made on this basis. However, the bottleneck and main criticism of using this approach is that it always relies on some arbitrary assumptions to define the slipping plane location (e.g., based on water density profiles, etc.).

Recently, Předota and co-workers^1^ revisited the NEMD simulation framework where ZP values are derived from limiting EO velocities, with no need for arbitrary assumptions about the shear plane position. The relation between EO mobility at distance *z* from the surface, μ_*x*_^eo^(*z*), as defined in eq 2, and the potential at the shear plane (ζ) can be given by the Smoluchowski formula, which is derived from a more general formula^41^ and reads as follows
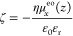
3in which ε_0_ is the vacuum permittivity, ε_r_ is the relative dielectric constant, and η is the coefficient of viscosity, for which either water experimental (Table 3) or water models’ values (Table S1) have been used.^42^ The negative sign indicates that when ζ is negative the EO flow has the same direction of the electric field. The relation in eq 3 is valid under the conditions that the particle radius is so large that the double layer can be considered flat, i.e., when the curvature radius *a* is much larger than the Debye length κ^–1^ (κ*a* ≫ 1), and the liquid from the shear plane towards the bulk phase is not affected by the applied electric field.

**Table 3:** ZP Values Estimated from the Average EO Mobility of Flexible Bulk-Water Molecules at 0.15 or 0.30 M NaCl Concentration Using Either Full-3D Periodic (P3M) or Slab Correction (P3M-SC) Treatment^a^

ζ (mV)	0.10 M	0.15 M (P3M)	0.15 M (P3M-SC)	0.30 M (P3M)	0.30 M (P3M-SC)	0.50 M
TIP3P-FB		–9.2 ± 0.7 (+13.2 ± 0.8)	–12.6 ± 0.7 (−5.5 ± 0.6)	+9.1 ± 0.7 (+12.1 ± 0.7)	–5.2 ± 0.6 (+12.0 ± 0.7)	
TIP3Pm		–27.2 ± 1.2 (+30.7 ± 0.9)	–29.0 ± 0.8 (+17.5 ± 1.0)	+2.1 ± 0.7 (+39.9 ± 0.9)	+11.7 ± 0.8 (+29.0 ± 1.0)	
TIP4P-FB		–4.9 ± 1.3 (+26.7 ± 1.3)	–15.9 ± 0.8 (+23.4 ± 1.8)	+53.1 ± 2.6 (+74.8 ± 2.5)	+38.0 ± 1.4 (+94.1 ± 1.9)	
TIP4P-Ew		–11.3 ± 1.6 (+30.0 ± 2.5)	–12.8 ± 1.0 (+20.1 ± 1.5)	+92.1 ± 3.1 (+109.3 ± 3.7)	+83.4 ± 2.6 (+122.5 ± 2.5)	
TIP4P/2005		–134 ± 2.8 (−72.3 ± 1.1)	–58.3 ± 1.3 (−32.1 ± 1.5)	–118 ± 0.9 (−55.1 ± 1.0)	–31.1 ± 1.6 (−29.6 ± 1.8)	
Exp.	–13.0^11^	–12.5 ± 0.9^†^		–5.8 ± 0.7^†^		–0.5^11^

a The ZP values predicted by the rigid models are given in parenthesis. The ZP values are estimated from the EO mobility averaged within 40 and 50 Å from the TiO_2_ slab center. Water experimental values of dielectric constant and coefficient of viscosity have been used (79 for ε_r_ and 8.9 × 10^–4^ Pa S for η). Experimental ZP values were measured with ELS (private communication, indicated with †) or electroacoustic^11^ experiments.

Table 2 reports the ZP values from the limiting EO mobility of water molecules within the region depicted by the two dashed lines in Figures 2 and 3 (between 40 and 50 Å from the TiO_2_ slab) predicted by the five TIP*x*P FFs benchmarked in Section 3.2, using either flexible or rigid water molecules. An alternative ZP analysis can be found in Table S1, where we use the corresponding dielectric constant and viscosity for every TIP*x*P water model estimated in pure and 0.15 M NaCl solutions, respectively.

Of particular interest for this study is the quantitative comparison between NEMD predictions from this computational study and the corresponding experimental values of ZP for anatase TiO_2_ NPs in NaCl solutions at neutral pH. In pH-controlled ELS experiments, the ZP of anatase TiO_2_ NPs at neutral pH remains negative from 0.15 to 0.30 M NaCl concentration (Table 3), varying from −12.5 to −5.8 mV, respectively (private communication). Similarly, electroacoustic experiments also predict negative ZP values for TiO_2_ NPs in an aqueous solution at a similar range of NaCl concentration (0.10–0.50 M) and pH conditions.^11^ Hence, we adopt the following criteria to judge the accuracy of different NEMD simulation setups to determine the ZP: (i) overall magnitude of ZP values; (ii) qualitative prediction of ZP sign and experimental trends; and (iii) quantitative agreement of simulated vs experimental ZP values.

Data reported in Table 3 confirm the reliability of the NEMD method to predict the magnitude of ZP values (∼mV) correctly. Indeed, the straightforward approach based on the analysis of electrostatic potential profiles to determine ZP (Figure S11, Supporting Information), for which an ill-defined shear plane has been assumed as done in ref (43), yields to deviations at least one order of magnitude larger (∼V) (Table S2, Supporting Information) compared to the former approach. Another important feature of our NEMD results (Table 3) is the ZP values behavior upon adding salt to the solution. In line with the expected textbook behavior, we find that ZP changes towards less-negative values upon increasing NaCl in the solution.

Next, we call attention to the crucial role of the intramolecular motion of water in determining ZP values of negatively charged anatase TiO_2_ NPs. Table 3 reveals the overall poor performance of rigid models in predicting ZP values at 0.15 or 0.30 M of NaCl in solution. With a few exceptions in Table 3, the use of rigid solvent molecules always yields positive ZP values (negative EO mobilities, Section 3.2), in disagreement with most experimental findings reported in the literature.^11,44−52^

Surprisingly, by only swapping the intramolecular treatment of water molecules from rigid to flexible, all FFs predict the sign of ZP in qualitative accordance with the experimental observations at 0.15 M of NaCl in solution. Some simulation setups (Table 3) can even quantitatively predict the experimental data of ZP at 0.15 M of NaCl, although one must keep in mind that the ZP values directly depend on the choice of dielectric and viscosity inputs (Table 3 vs Table S1). At 0.30 M of NaCl concentration, although the introduction of water flexibility improves the ZP prediction compared to the rigid models, most combinations between FFs and simulation setups tested here fail to predict the correct sign of ZP. The only exceptions are the combinations between flexible TIP3P-FB and P3M-SC, and TIP4P/2005 under either P3M or P3M-SC treatment, with the former setup predicting the ZP value quantitatively at 0.30 M of NaCl.

Overall, we demonstrate that water flexibility strongly impacts the ZP prediction. The inclusion of water flexibility enhances the agreement between our numerical predictions and the experimental measurements of ZP for anatase TiO_2_ NPs in an aqueous solution at 0.15 or 0.30 M of NaCl concentration under neutral pH conditions. In particular, we find that flexible TIP3P-FB outperforms other combinations between FFs and simulation protocols tested here. Both rigid and flexible TIP4P/2005, although catching the correct ZP sign, underestimate the numeric value of ZP compared to experiments.

## 4 Conclusions

NEMD methods have stood out as a powerful tool to investigate the electrokinetic phenomena of NPs in solution and highly rely on the quality of available empirical FF parameters and appropriate simulation protocols. However, the impact of solvent modeling on NEMD predictions is still overlooked. Thus, there is a call for systematical benchmarking studies of available FFs, and the search for best practice protocols to achieve superior simulation outcomes should be a priority.

Our study clarifies how critical simulation parameters and solvent modeling influence the electrodynamic properties of saline solutions interfacing with a realistic hydroxylated TiO_2_ (101) anatase surface at neutral pH. Based on a systematic comparison of five mainstream TIP*x*P FFs for water and ions, our results reveal the essential role of water flexibility in correctly predicting EO mobilities, and hence, determining ZP values.

We demonstrate that removing degrees of freedom by constraining water bonds and angles to their equilibrium lengths and angular values yields poor performance of NEMD simulations in reproducing experimental ZP data. The rigid-geometry approximation for solvent molecules enhances the condensation and adsorption of co- and counter-ions at the interfacial region, in most cases, leading to EO flow reversal. We attribute this behavior to the lower dielectric constant of aqueous solutions when water molecules’ internal degrees of freedom are removed, likely strengthening the electrostatic interactions between ions and TiO_2_ surface atoms.

By introducing bending degrees of freedom in water molecules, making them flexible, we observe the enhanced performance of our NEMD calculations in accurately determining the experimental ZP values for anatase TiO_2_ NPs at moderate NaCl concentration. Among the investigated water/ion TIP*x*P FFs, we found that TIP3P-FB (in combination with P3M-SC electrostatic treatment) and TIP4P/2005 can correctly predict the ZP sign at 0.15 or 0.30 M of NaCl in solution, with the former model even showing fair agreement with the experimental values of ZP at these two salt regimes.

These findings provide practical simulation guidelines for modeling ZP of nano- or micro-sized TiO_2_ particles in NaCl solution using the NEMD method. Additionally, we envision that the methodological guidance proposed herein applies not only to solid/liquid interfaces but can also be extended over a broader class of interfacial systems where solvent modeling plays a crucial role (e.g., biomembranes/water).

## Supporting Information Available

The choice of electric field strength; the influence of reflective walls on the water and ion distribution; NaCl RDF profiles for different water/ion FFs; probability of Na^+^ and Cl^–^ ions near the TiO_2_ surface; number density profiles of co- and counter-ions normal to the TiO_2_ surface under P3M-SC treatment; EO mobility and surface charge screening profiles for TIP4P/2005/Madrid 2019 FF; bulk-water viscosity for the TIP*x*P water models in their rigid and flexible version; comparison of fluid viscosity, screening, and number density profiles between TIP4P-Ew and TIP4P/2005 models; alternative zeta potential estimations; and theoretical background for the estimation of fluid viscosity and dielectric constant (PDF)
