# Supplementary material for: Modeling Zeta Potential for Nanoparticles in Solution: Water Flexibility Matters
Source: J Phys Chem C Nanomater Interfaces. 2023 May 9;127(19):9236–47. doi: 10.1021/acs.jpcc.2c08988 (PMC10201526; doi:10.1021/acs.jpcc.2c08988)
Supplement: Supplementary file 1 — jp2c08988_si_001.pdf [file jp2c08988_si_001.pdf]

Supporting Information

Modeling Zeta Potential for Nanoparticles in Solution: Water Flexibility Matters

Paulo Siani,[a] Giulia Frigerio,[a] Edoardo Donadoni,[a] Cristiana Di Valentin[a,*]

[a]*Dipartimento di Scienza dei Materiali, Università di Milano Bicocca, via R. Cozzi 55, 20125 Milano, Italy; BioNanoMedicine Center NANOMIB, University of Milano-Bicocca, Italy.*

*Corresponding author: cristiana.divalentin@unimib.it

Section S1. The choice of electric field strength

Upon analysis of Figure S1, we notice that the electric field strength of 0.02 V/Å impacts mostly the first layer of Na^+ ions adsorbed on the negatively charged TiO_2 surface while the distribution of Na^+ ions in the diffuse layer is less affected by it. Noteworthy, the difference in the first peak intensity between EMD and NEMD simulations corresponds to less than 2 Na^+ ions being removed from the TiO_2 surface by the action of the external electric field. Therefore, we conclude that for this specific system setup (highly negatively charged TiO_2 surface at moderate salt concentration) the chosen electric field strength provides fair performance and keeps an acceptable response on the stream velocity of water. Further, we observe little disturbance compared to the distribution of Na^+ ions from the EMD simulations. One should be aware that other systems (e.g., neutral or barely charged surface interfacing with an aqueous solution) may behave differently and the impact of a particular electric field strength has to be verified beforehand. Figure S1 shows the ion density profile of Na^+ ions normal to the TiO_2 surface in the presence (NEMD simulation) or absence (EMD simulation) of an external electric field.

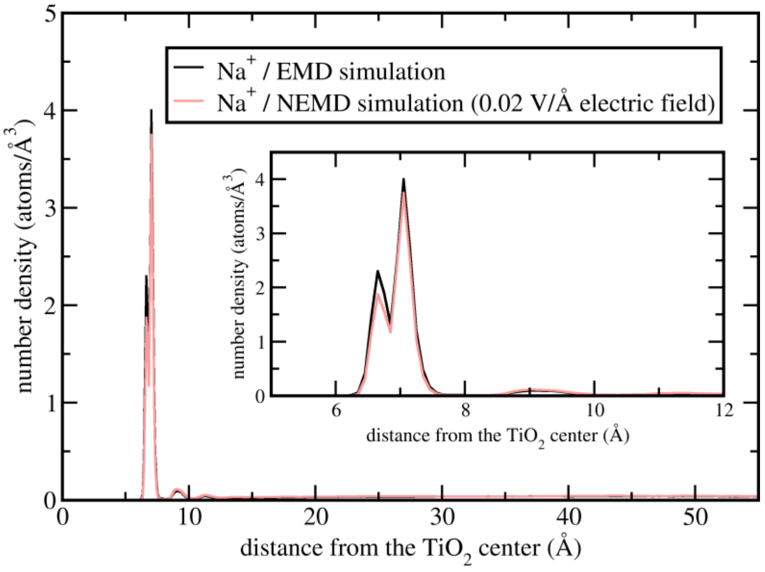

Figure S1. Number density profiles of Na^+ ions normal to the TiO_2 slab in the presence (orange) or absence (black) of an external electric field.

Section S2. The influence of reflective walls on the water and ion distribution

Upon analysis of the water density profiles in Figure S2, we identify that the water density is kept at its bulk value up to 52.5 Å from the TiO_2 slab center, evidenced by a clear plateau in the water density profiles, whether the reflective walls are present or not. In both cases, we identify that the bulk-water density starts consistently dropping from 55 Å outwards, suggesting the formation of a liquid-vapor interface. In the presence of reflective walls, we identify that this liquid-vacuum interface takes place at closer distances from the TiO_2 surface due to the presence of reflective walls. While for the simulation without reflective walls at the z-boundaries, we notice that the liquid-vapor region becomes more extensive outwards compared to the simulation setup where the particle confinement is imposed at the z-boundaries.

Moreover, we notice that the ion density starts deviating from its bulk-like value at shorter distances from the TiO_2 surface than the water density does. Figure S2 shows that the bulk-like behavior in the ion density profiles, either in the presence or absence of reflective walls, is lost at distances farther than ~50 Å from the TiO_2 slab center. Importantly, we do not identify any significant deviation in the ion concentration due to the placement of reflective walls, but a slightly lower ion density in their absence between 40 and 50 Å from the TiO_2 slab center. Hence, we argue that the lowering in the water and ion density near the edge of the simulation box is caused by the formation of a liquid-vapor interface at the z-boundaries rather than simulation artifacts arising from spurious interaction between ions and the reflective walls.

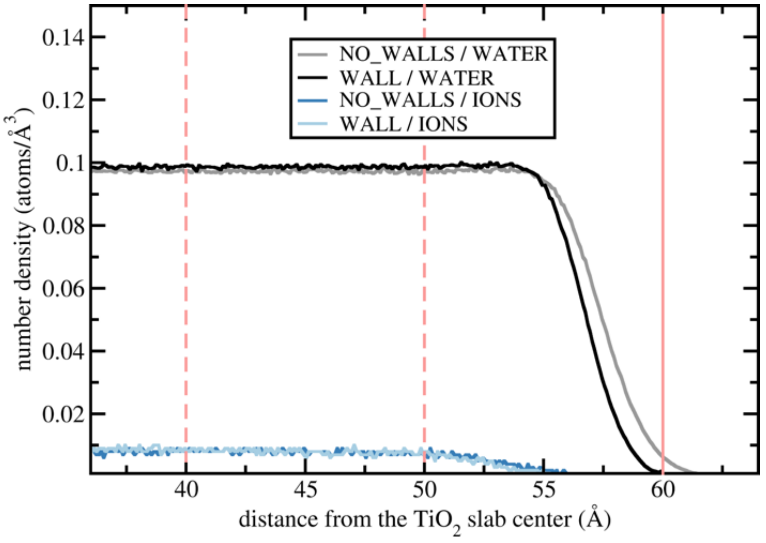

Figure S2. 1D number density profile for water and ions along the z-direction to the TiO_2 slab in the presence and absence of reflective walls. Color code: water density with reflective walls (black curve); water density without reflective wall (grey curve); ion density with reflective walls (dark blue); ion density without reflective walls (light blue); region used for zeta potential estimation (region within the two dashed red lines); reflective walls (solid red line).

Section S3. Na^+Cl^- RDF profiles for different water/ion FFs

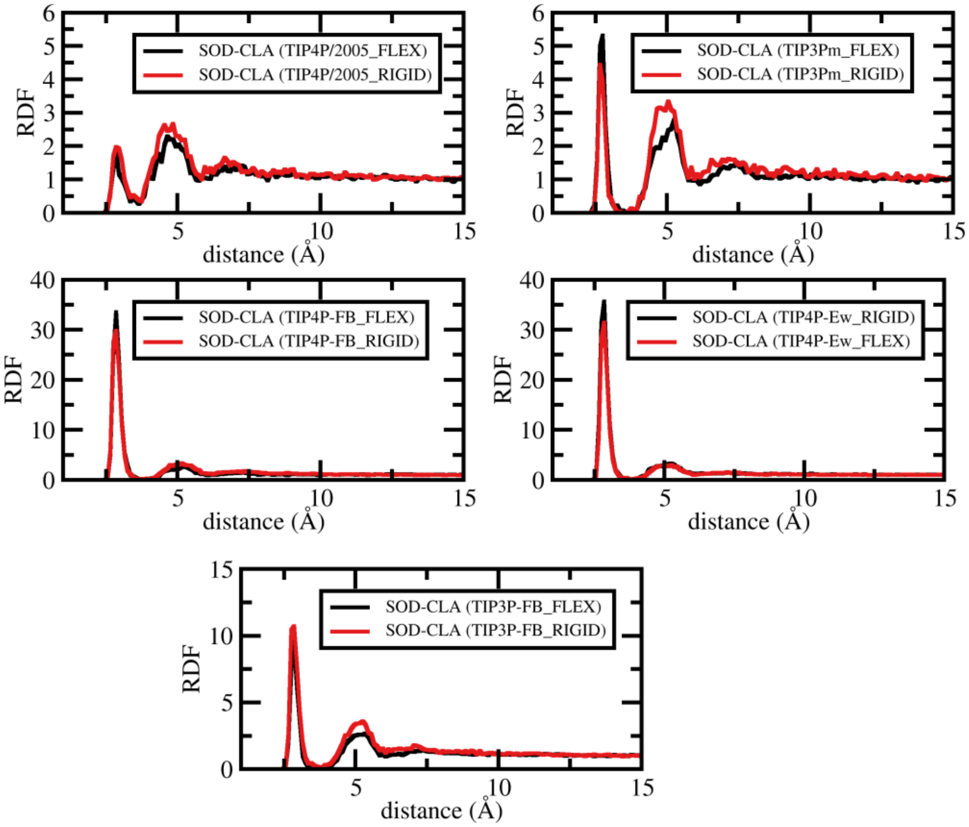

Figure S3. Na^+Cl^- RDF profiles for the different water/ion force fields adopted in this work.

Section S4. Probability of Na^+ and Cl^- ions near the TiO_2 surface

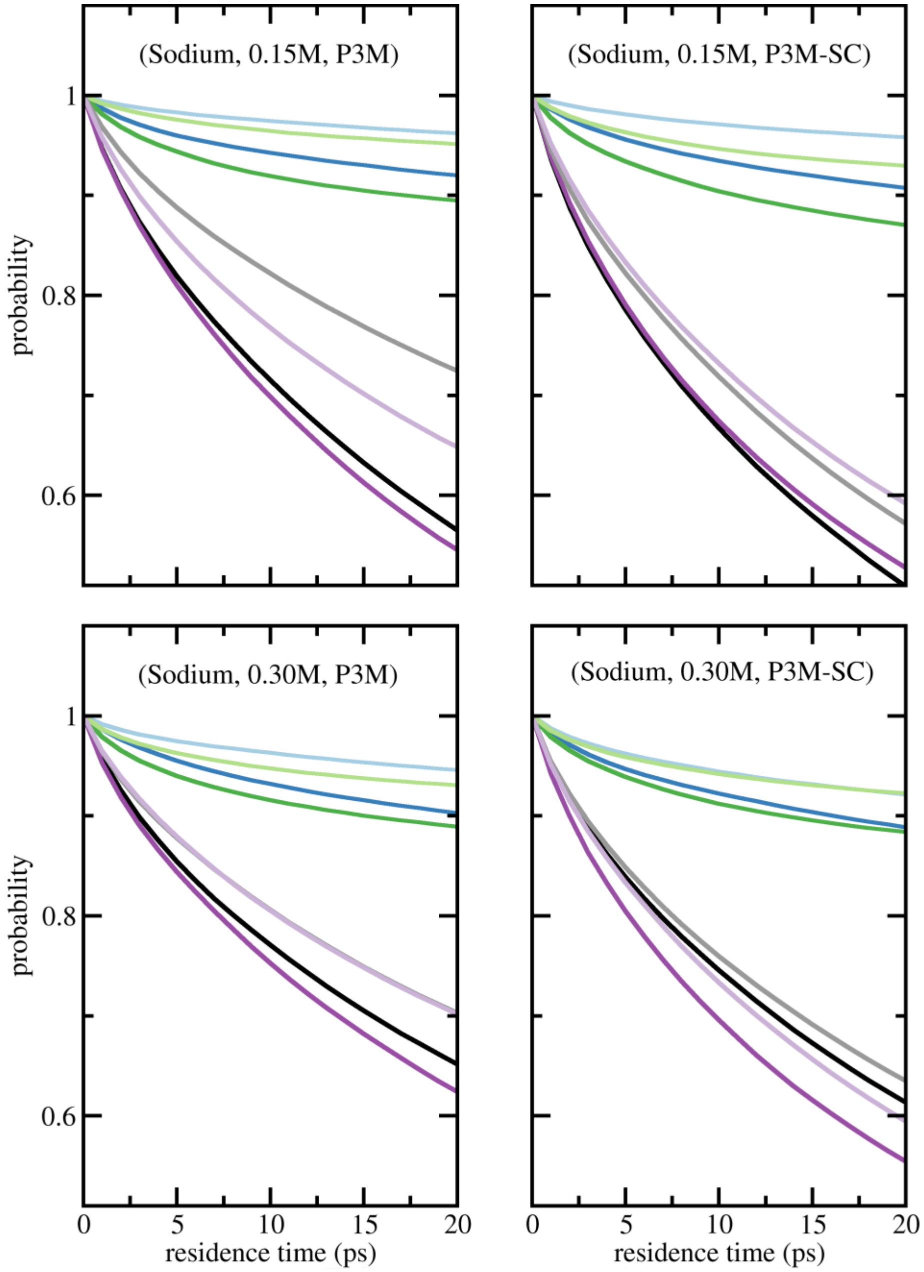

Figure S4. Probability of finding Na^+ counter-ions within 7.5 Å normal to the TiO_2 surface under P3M or P3M-SC at 0.15 M or 0.30 M of NaCl in solution. Color codes: (blue) TIP3P-FB FF and flexible model, (light blue) TIP3P-FB FF and rigid model, (green) TIP3Pm FF and flexible model, (light green) TIP3Pm FF and rigid model, (black) TIP4P-FB FF and flexible model, (grey) TIP4P-FB FF and rigid model, (violet) TIP4P-Ew FF and flexible model, and (light violet) TIP4P-Ew FF and rigid model.

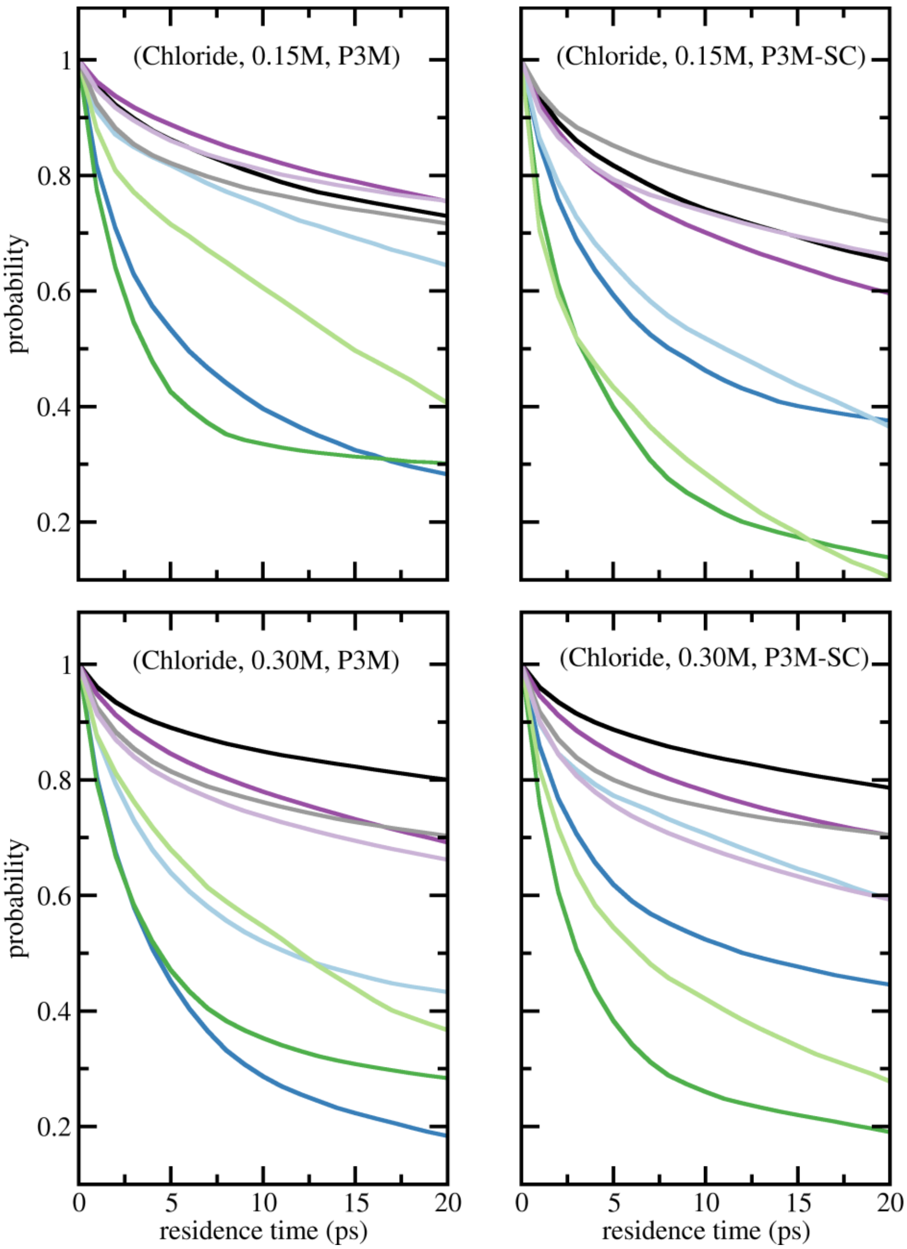

Figure S5. Probability of finding Cl^- co-ions within 7.5 Å normal to the TiO_2 surface under P3M or P3M-SC at 0.15 M or 0.30 M of NaCl in solution. Color codes: (blue) TIP3P-FB FF and flexible model, (light blue) TIP3P-FB FF and rigid model, (green) TIP3Pm FF and flexible model, (light green) TIP3Pm FF and rigid model, (black) TIP4P-FB FF and flexible model, (grey) TIP4P-FB FF and rigid model, (violet) TIP4P-Ew FF and flexible model, and (light violet) TIP4P-Ew FF and rigid model.

Section S5. Number density profiles of co- and counter-ions normal to TiO_2 surface under P3M-SC treatment

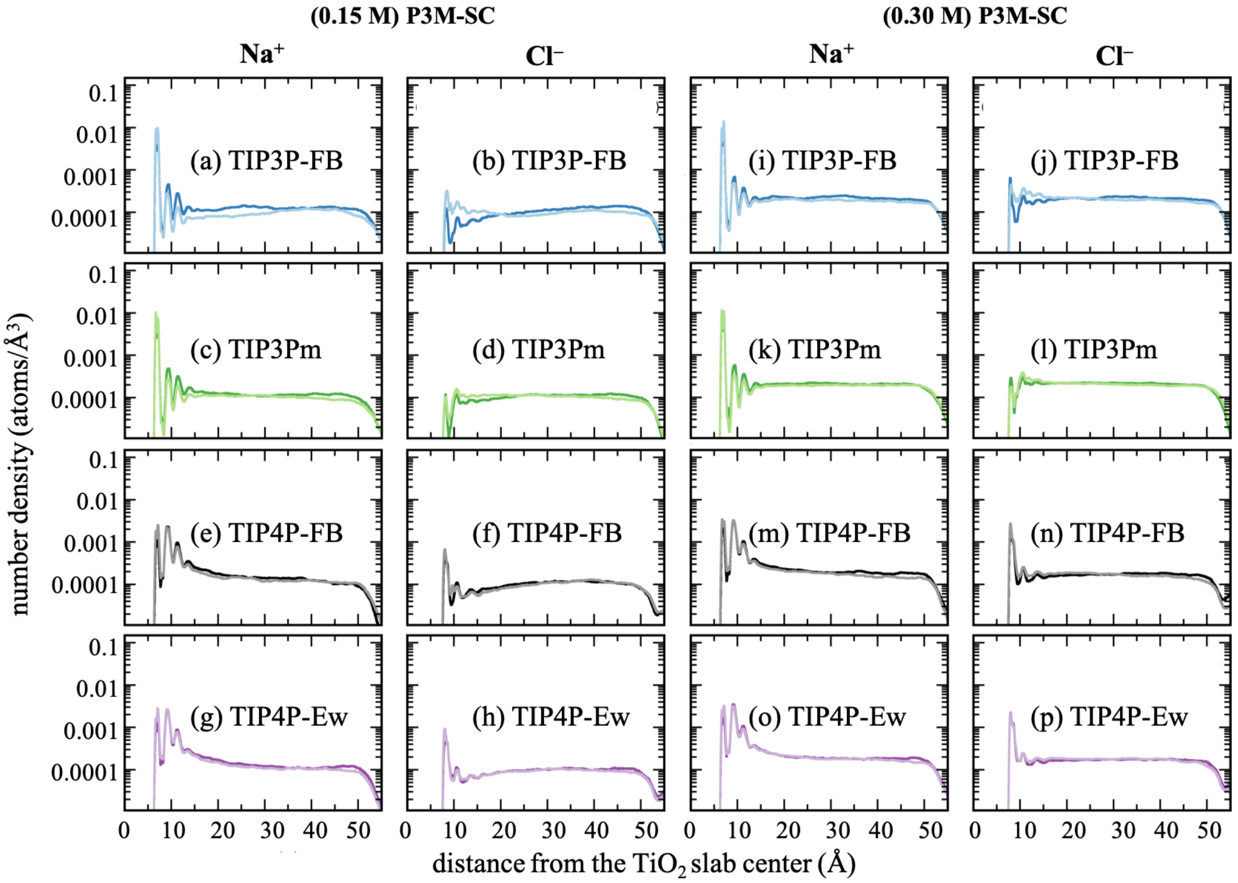

Figure S6. Number density profiles of co- and counter-ions normal to the negatively charged TiO_2 surface at 0.15 M (a-h) or 0.30 M (i-p) NaCl concentration under P3M-SC treatment. Color codes: (blue) TIP3P-FB and flexible model, (light blue) TIP3P-FB and rigid model, (green) TIP3Pm and flexible model, (light green) TIP3Pm and rigid model, (black) TIP4P-FB and flexible model, (grey) TIP4P-FB and rigid model, (violet) TIP4P-Ew and flexible model, and (light violet) TIP4P-Ew and rigid model.

Section S6. EO mobility and surface charge screening profiles for TIP4P/2005 FF in combination with Madrid 2019 FF for ions.

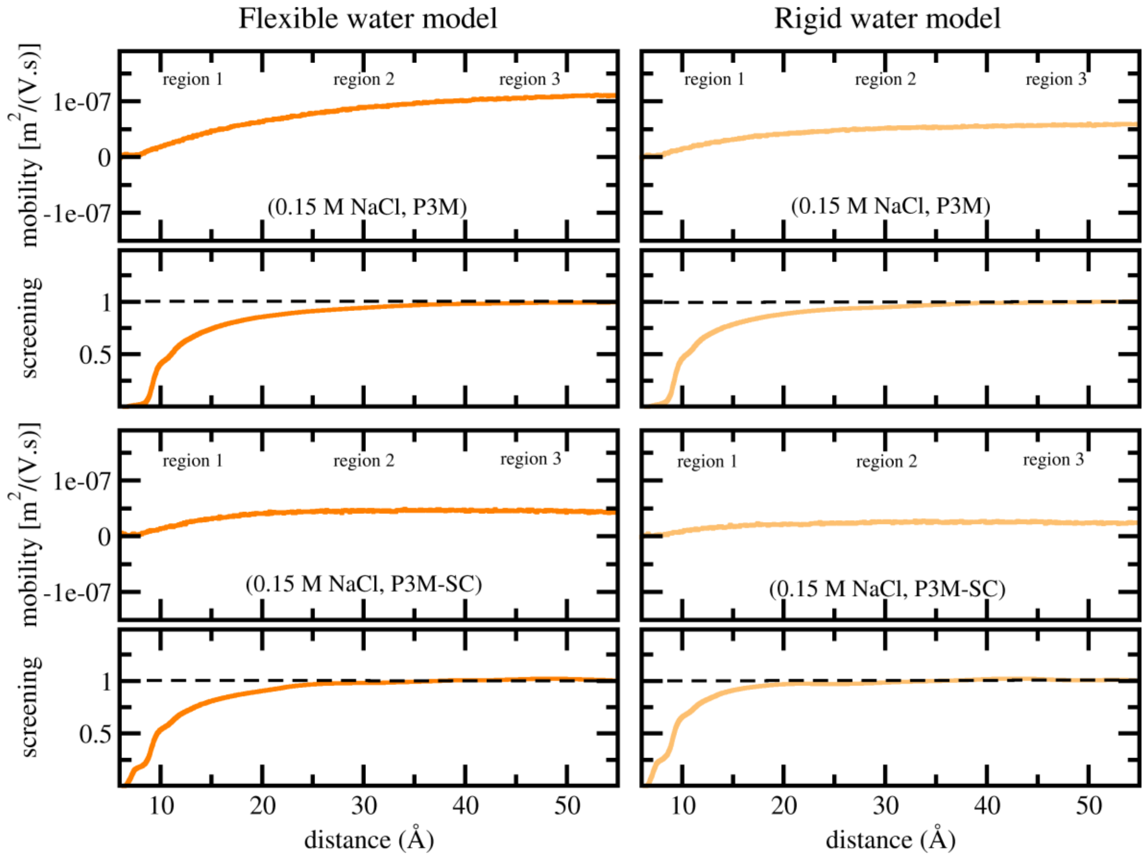

Figure S7. EO mobility and surface charge screening profiles of TIP4P-2005 water molecules normal to the negatively charged TiO_2 slab at 0.15 M of NaCl in aqueous solution under either regular P3M or P3M-SC electrostatic treatment.

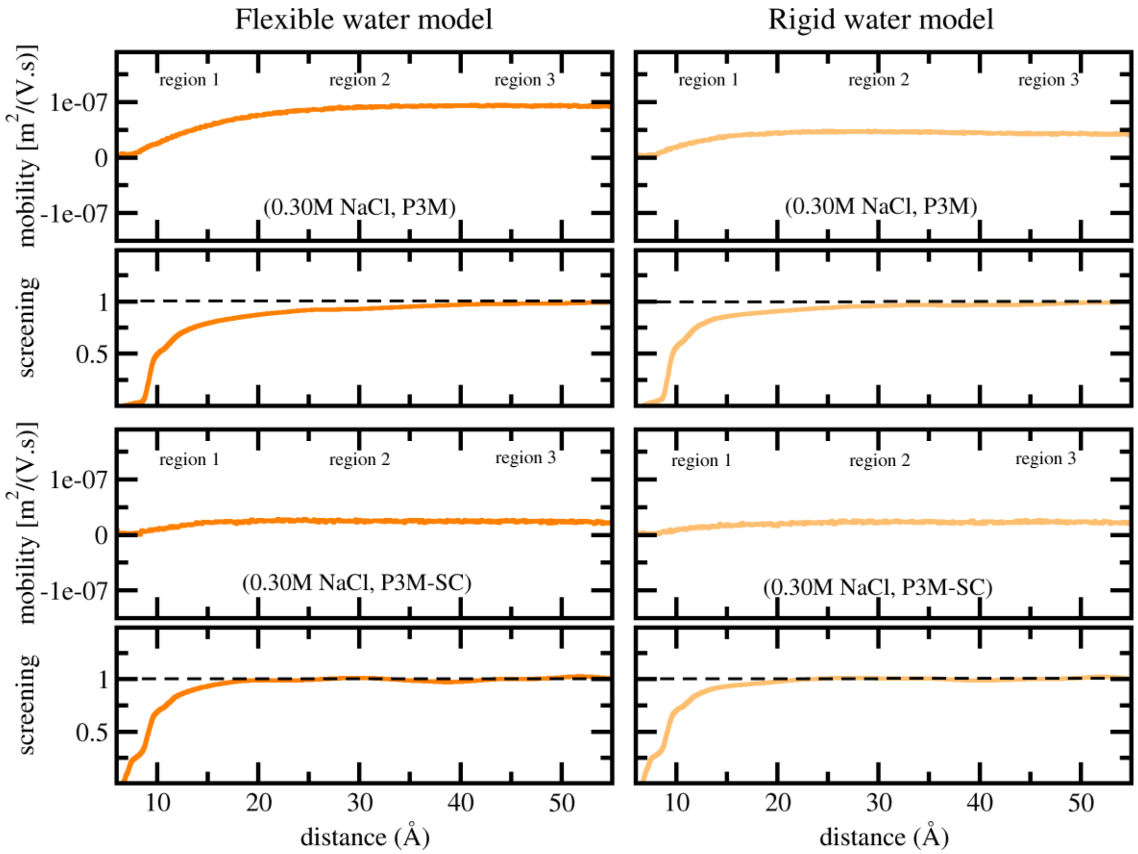

Figure S8. EO mobility and surface charge screening profiles of TIP4P-2005 water molecules normal to the negatively charged TiO_2 slab at 0.30 M of NaCl in aqueous solution under either regular P3M or P3M-SC electrostatic treatment.

Section S7. Bulk-water viscosity of TIPxP water models in their rigid or flexible version

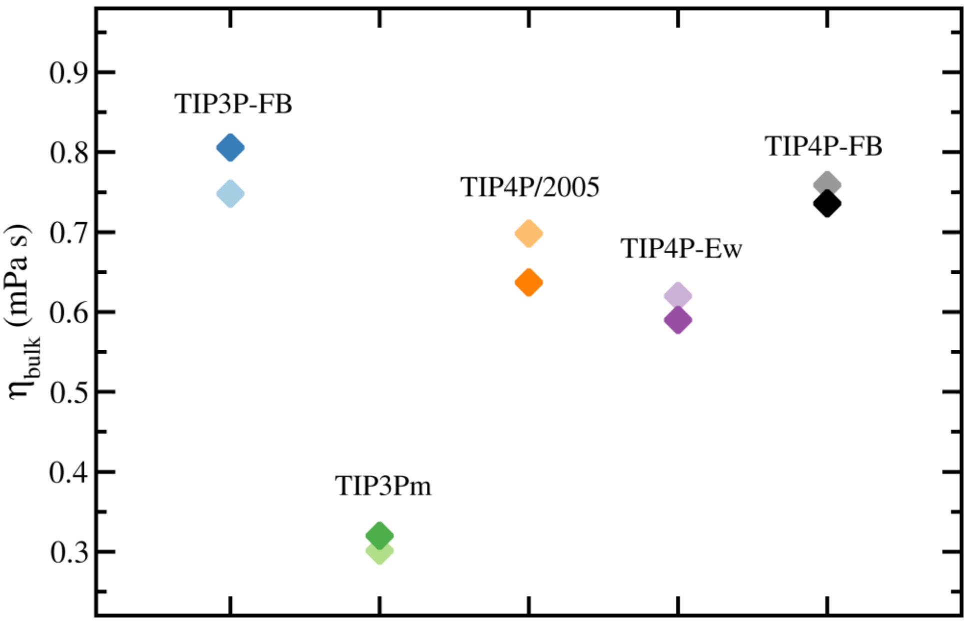

Figure S9. Bulk-water viscosity of aqueous salt solutions at 0.15 M of NaCl for every water/ion TIPxP FF studied. Solid and light colors represent the flexible and the rigid water models, respectively.

Upon analysis of Figure S9, we observe that introduction of water flexibility yields higher viscosity compared to their rigid version in TIP3P models, whereas TIP4P models behave otherwise. Thus, we find no clear correlation between the viscosity changes due to the presence or absence of water flexibility. Nonetheless, these data have been useful to carry out an alternative analysis of zeta potentials since the proper dielectric constant and viscosity for each water model can be utilized instead of standard experimental parameters.

Section S8. Comparison of fluid viscosity, screening and number density profiles between TIP4P-Ew and TIP4P/2005 models

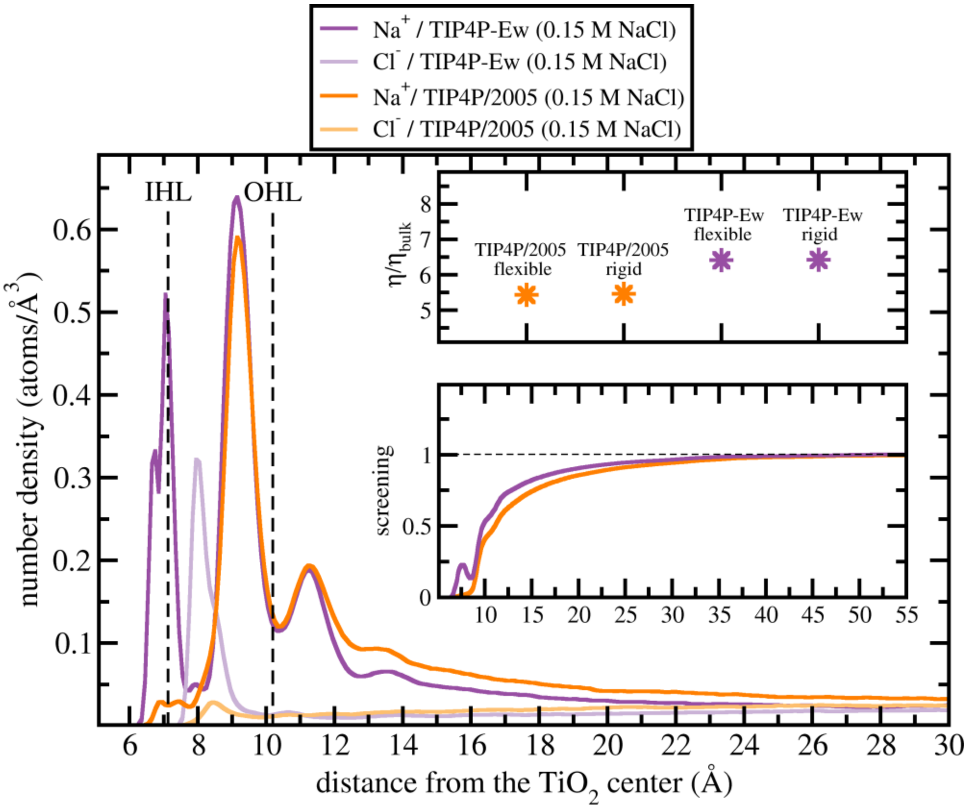

Figure S10. Number density profiles of Na^+ and Cl^- ions normal to the TiO_2 slab for TIP4P-Ew and TIP4P/2005 FFs using flexible water molecules. Top inset: Ratio between the viscosity values calculated for the water/ion solution in contact with the TiO_2 surface and the bulk viscosity calculated in separate simulations of the same solution in the absence of the TiO_2 slab for TIP4P-Ew and TIP4P/2005 FFs using flexible or rigid water model. Bottom inset: Surface charge screening for TIP4P-Ew and TIP4P/2005 using flexible water model. All the data refer to systems at 0.15 M of NaCl concentration.

Section S9. Alternative zeta potential estimations

Table S1. Alternative ZP estimation using the corresponding dielectric constant (Table 2), viscosity (Figure S9), and average EO mobility for every TIPxP water model. The average EO mobility from flexible or rigid water models, at 0.15 M or 0.30 M of NaCl concentration, using full-3D periodic (P3M) or slab geometry (P3M-SC). The ZP values predicted by the rigid models are given in parenthesis. Experimental ZP values were measured with ELS (private communication, indicated with †) or electroacoustic[24] experiments.

ζ(mV)	0.10 M	0.15 M (P3M)	0.15 M (P3M-SC)	0.30 M (P3M)	0.30 M (P3M-SC)	0.50 M
**TIP3P-FB**	-	-6.6±0.5 (12.1±0.7)	-9.0±0.5 (-5.1±0.6)	6.5±0.5 (11.1±0.6)	-3.7±0.5 (11.0±0.6)	-
**TIP3Pm**	-	-6.7±0.3 (9.5±0.3)	-7.2±0.2 (5.4±0.3)	0.5±0.2 (12.3±0.3)	2.9±0.2 (9.0±0.3)	-
**TIP4P-FB**	-	-4.0±1.1 (26.2±1.2)	-12.8±0.6 (22.9±1.8)	43.0±2.1 (73.5±2.4)	30.8±1.1 (92.4±1.9)	-
**TIP4P-Ew**	-	-9.0±1.3 (29.7±2.5)	-10.2±0.8 (19.9±1.4)	73.4±2.5 (108.0±3.6)	66.5±2.1 (121.0±2.4)	-
**TIP4P/2005**	-	-127.8±2.6 (-83.5±1.3)	-55.4±1.2 (-37.0±1.7)	-112.9.5±0.9 (-63.6±1.2)	-29.5±1.5 (-34.1±2.0)	-
**Exp.**	-13.0	-12.5±0.9†		-5.8±0.7†		-0.5

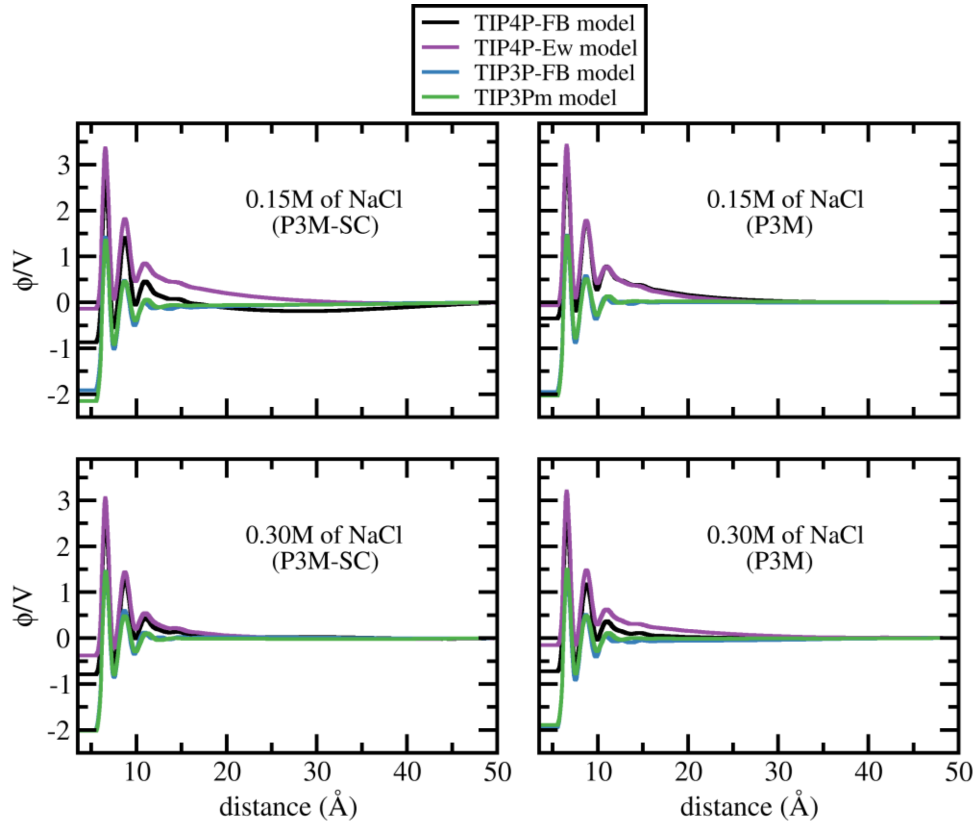

Figure S11. Electrostatic potential profiles of bulk-water salt solutions normal to the TiO_2 surface at 0.15 M or 0.30 M of NaCl in solution using flexible TIPxP models. Color codes: (blue) TIP3P-FB, (green) TIP3Pm, (black) TIP4P-FB, (violet) TIP4P-Ew.

Table S2. Electrostatic potential difference between the bulk-water phase and the shear plane (arbitrarily defined as the third minimum in the number density profiles in Figure S11) for flexible TIPxP-based models under P3M or P3M-SC treatment.

$\zeta(V)$	**0.15 M (P3M)**	**0.15 M (P3M-SC)**	**0.30 M (P3M)**	**0.30 M (P3M-SC)**
**TIP3P-FB**	-1.95	-1.91	-1.93	-2.00
**TIP3Pm**	-2.04	-2.15	-1.89	-2.02
**TIP4P-FB**	-0.35	-0.87	-0.72	-0.79
**TIP4P-Ew**	-0.06	-0.14	-0.15	-0.38

Section S10. Theoretical background for the estimation of fluid viscosity and dielectric constant

Fluid viscosity

Fluid viscosity was calculated using the periodic perturbation method[1]. Herein, an external force ***a*** is applied to produce a velocity field ***v*** in the liquid such that $\boldsymbol{a_y}$ and $\boldsymbol{a_z}$ are zero and $\boldsymbol{a_x}$ is a function of ***z*** only. To satisfy the system periodicity and obtain a smooth and periodic velocity profile with small local shear rates, an acceleration given by a periodic cosine function is applied, which can be expressed as:

$$a_x(z) = A\cos(kz) \quad \text{(Eq. S1)}$$

$$k = \frac{2\pi}{l_z} \quad \text{(Eq. S2)}$$

in which A is the acceleration amplitude (herein 0.02e-5 Å/fs^2) and l_z is the z-length of the simulation box. At steady state, the velocity profile generated by this acceleration is

$$v_x(z) = V\cos(kz) \quad \text{(Eq. S3)}$$

where V is the generated cosine-shaped velocity amplitude and its ensemble average is related to the shear viscosity accordingly to

$$\eta = \frac{A}{V}\frac{\rho}{k^2} \quad \text{(Eq. S4)}$$

in which ρ is the fluid density, η is the shear viscosity and V the cosine-shaped velocity amplitude. The ensemble average of V is taken from the velocity profile via

$$V(t) = \frac{2\sum_{i=1}^{N} m_i v_{i,x}(t)\cos\left(kr_{i,z}(t)\right)}{\sum_{i=1}^{N} m_i} \quad \text{(Eq. S5)}$$

where m_i , $v_{i,x}$ and z_i are the atomic mass, x-component of velocity, and z-coordinate of the particles, respectively.

Dielectric constant

The static dielectric constant was estimated by analyzing the fluctuation in the total dipole moment of pure water systems composed of TIPxP models in their rigid or flexible version, using the following relation:

$$\varepsilon_{bulk} = 1 + \frac{\langle M^2 \rangle - \langle M \rangle^2}{3\varepsilon_0 V k_b T} \quad \text{(Eq. S6)}$$

where V stands for the volume of the simulation box, k_b is the Boltzmann constant, and $\langle M^2 \rangle - \langle M \rangle^2$ the directional dipole moment fluctuation in $(e\text{Å})^2$.

References

(1) Hess, B. Determining the shear viscosity of model liquids from molecular dynamics simulations. *The Journal of Chemical Physics* **2002**, *116* (1), 209-217.
